# Natural products target programmed cell death signaling mechanisms to treat colorectal cancer

**DOI:** 10.3389/fphar.2025.1565332

**Published:** 2025-04-24

**Authors:** Ya Zheng, Na Feng, Canglin Li, Zuoqiang Li

**Affiliations:** ^1^ The Second Gastroenterology Department, Affiliated Hospital of Shandong University of Traditional Chinese Medicine, Jinan, China; ^2^ Department of Rehabilitation Medicine, Linyi Maternal and Child Health Center Hospital, Linyi, Shandong, China; ^3^ Medical Management Department, Affiliated Hospital of Shandong University of Traditional Chinese Medicine, Jinan, China; ^4^ Department of Traditional Chinese Medicine, Shandong Academy of Occupational Health and Occupational Medicine, Jinan, China

**Keywords:** CRC, PCD, natural product, mechanism, Chinese medicine, drug resistance

## Abstract

As a highly prevalent gastrointestinal malignant tumor, colorectal cancer poses a serious challenge in terms of increasing morbidity and mortality and late diagnosis due to the invisibility of the disease. Although existing therapies are diverse but limited in efficacy, the mechanism of programmed cell death (PCD) has become a focus of research due to its central role in maintaining body homeostasis and regulating tumor progression. Multimodal cell death pathways, such as apoptosis, autophagy, pyroptosis and ferroptosis, have shown unique advantages in inhibiting the proliferation and migration of colorectal cancer cells and enhancing the sensitivity to chemotherapy by responding to internal and external environmental stimuli. In recent years, natural products have risen to prominence by virtue of their multi-target synergistic effects and chemo-sensitizing properties, and have opened up a new direction for colorectal cancer treatment by precisely regulating the PCD pathway. In this paper, we searched PubMed, Web of Science and CNKI databases for relevant studies in the last 10 years using the keywords (Colorectal cancer) and (programmed cell death) and natural products. This work retrieved 59 studies (55 from the past 5 years and 4 from the past 10 years) to reveal the mechanism of action of natural products targeting PCD, aiming to provide theoretical support and practical guidance for the optimization of clinical therapeutic strategies and the development of innovative drugs.

## 1 Introduction

Colorectal cancer (CRC) is a malignant tumor originating from the colon and intestinal epithelial cells, the mechanism of which is still unclear and may be related to inflammation, hypoxia, immunity, genetics, and aberrant activation of tumor-associated signaling pathways (Dekker et al., 2019). Clinical symptoms of CRC mainly include abdominal pain, hemorrhage in the stool, intestinal obstruction, abdominal mass etc., and even the appearance of metastatic lesions in other organs such as liver and lung. It has been reported that the incidence and mortality of CRC are increasing worldwide, ranking 3rd in the global cancer incidence rate, accounting for about 10%, and 2nd in the case fatality rate, accounting for about 9.4%, which undoubtedly creates a strong challenge to the global healthcare and economy (Sung et al., 2021).

**FIGURE 1:**
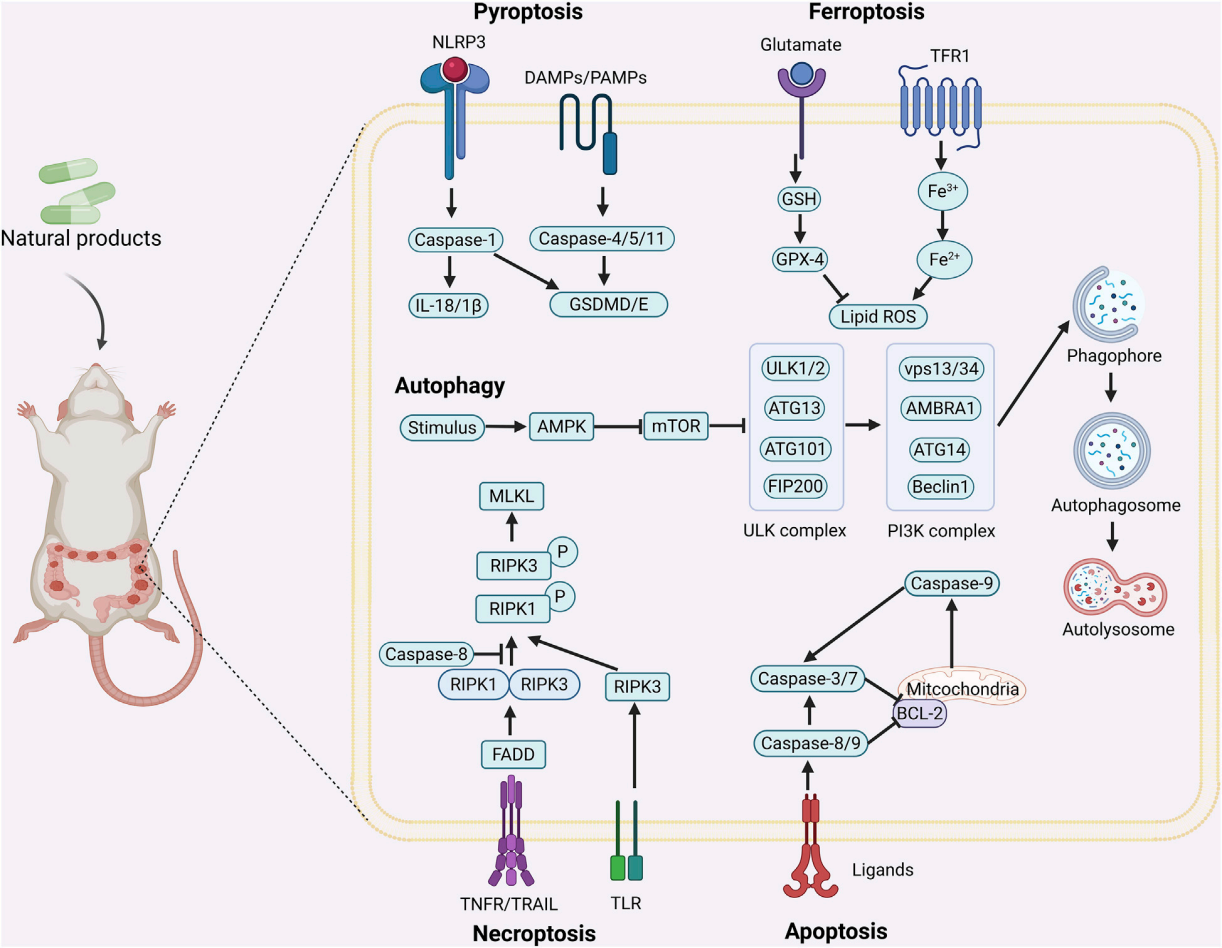
Mechanism of action of natural product-targeted modulation of PCD for the treatment of CRC. Apoptosis is regulated by internal and external pathways, the Bcl-2 family regulates the release of cytochrome c from mitochondria and activates Caspase-9 and Caspase-3/7; the exogenous pathway promotes cell death by activating Caspase-3/7 through Caspase-8. Autophagy is the response of the ULK1 complex (ULK1/Atg13/FIP200) to nutrient deficiency by phosphorylating Beclin1; the latter forms the Vps34-Atg6 complex with Atg14, which drives nucleation of the autophagosome membrane and completes autophagosome formation. Ferroptosis is iron-dependent cell death, where ROS excess triggers lipid peroxidation via Fe^2+^ catalysis, GSH and GPX-4 antioxidant deficiencies, and Fe^2+^ to Fe^3+^ conversion led to cell death. Pyroptosis is triggered by NLRP3 activation of caspase-1/18, which shears GSDMD and forms pores that trigger cell rupture and inflammatory responses. Necroptosis is a receptor-mediated cell death mechanism mediated by RIPK1, RIPK3. RIPK1 binds to FADD to activate caspase-8, which induces apoptosis; under specific conditions, RIPK3 activates MLKL, which leads to rupture of the cell membrane and triggers necroptotic cell death. Created with Biorender.com.

Currently, surgery, radiotherapy, chemotherapy, immunotherapy, and targeted therapy are the main treatments for CRC, but problems such as the sequelae of surgery, chemotherapy resistance, toxic side effects, high metastasis and recurrence rates seriously affect the patients’ quality of life (Shin et al., 2023). Therefore, it is of great importance to find new therapeutic strategies for the treatment of CRC. Programmed cell death (PCD) is a kind of active and orderly cell death that is regulated by specific intracellular genes in response to a variety of stimuli, and has multiple pathways, including apoptosis, pyroptosis, necroptosis, autophagy, ferroptosis, etc., (Figure 1). The role of PCD is crucial in maintaining homeostasis of normal tissues, and there is a cross-interaction between multiple PCD modes, which together affect cellular outcomes (Yu et al., 2022). A large amount of data suggests that the occurrence and development of CRC are related to the aberrant expression of PCD and affect the regression and prognosis of CRC, which implies that targeting and regulating PCD may be able to bring new hope to CRC patients (Fan et al., 2021).

In recent years, natural products have shown great therapeutic potential and have been widely used in the treatment of a variety of oncological diseases, including hepatocellular carcinoma, gastric cancer, and breast cancer (Naeem et al., 2022). These products can exert anti-tumor effects in multiple targets and pathways, while ameliorating the toxic side effects caused by surgical chemotherapy, radiotherapy, targeted therapy and immunotherapy, and prolonging the survival time of patients (Micale et al., 2021). Excitingly, some natural products are candidates for the treatment of CRC, exerting anti-CRC tumor cell viability and proliferation by modulating different forms of PCD to inhibit CRC progression (Islam et al., 2022). Therefore, these natural products may become complementary and alternative therapies for CRC.

## 2 Natural products targeting programmed death in CRC

### 2.1 Apoptosis

Apoptosis is an active, orderly process of cell death that occurs under physiological or pathological conditions in order to maintain the homeostasis of its own internal environment through the activation, expression and regulation of a series of genes (Xu et al., 2019). This process involves a variety of morphological and biochemical changes, such as nuclear consolidation, DNA fragmentation, cell membrane remodeling and vesiculation, cellular crumpling, and the formation of apoptotic vesicles. The process can usually take place through three different pathways, namely the exogenous pathway, the endogenous pathway, and the endoplasmic reticulum stress-induced pathway (D'Arcy, 2019).

The exogenous pathway is triggered by the binding of ligands to transmembrane death receptors, and these death ligands mainly include FasL, tumor necrosis factor-α (TNF-α), TRAIL (TNF-related apoptosis-inducing ligand), etc., (Kashyap et al., 2021). In addition, FADD acts as a Fas-associated death domain protein, also known as Mort1. It has the ability to bind specifically to the cytoplasmic region of Fas and plays an interruptive role in apoptotic signaling. When Fas, also known as CD95, binds to the corresponding ligand, FasL, CD95L, the Fas receptor trimerizes and activates, and the activated receptor then binds to FADD, which in turn interacts with caspase-8, activating the latter to form a death-inducing signaling complex. This complex further activates a series of caspase proteins, such as caspase-1/3/7, etc., which promotes the onset of apoptosis in the cells where Fas proteins are located (Ketelut, 2022; Newton et al., 2024).

The endogenous pathway, also known as the mitochondria-mediated apoptosis pathway, is triggered by damage or stress signals within the cell (Kashyap et al., 2021). When a cell is damaged internally, it activates pro-apoptotic proteins in the B-cell lymphoma-2 (Bcl-2) family, such as Bad. These proteins alter the permeability of the outer mitochondrial membrane, resulting in the release of apoptosis-associated proteins, such as cytochrome c, from the mitochondrial membrane gap into the cytoplasm. The released cytochrome C binds to Apaf-1 protein in the cytoplasm to form apoptosome, which in turn activates Caspase-9 and activates downstream effector caspases, such as Caspase-3, to trigger apoptosis.

The endoplasmic reticulum stress pathway refers to the accumulation and aggregation of unfolded proteins in the presence of viral infections and calcium homeostasis disorders, leading to severe endoplasmic reticulum stress. This pathway not only induces the expression of endoplasmic reticulum molecular chaperones such as glucose-regulated proteins GRP78 and GRP94 to produce protective effects, but also independently induces endogenous apoptosis, which ultimately affects the regression of the stressed cells, such as adaptation, injury, or apoptosis (Suraweera et al., 2022; Kaloni et al., 2023).

Numerous data have demonstrated that targeting and regulating apoptosis can effectively inhibit the viability and proliferation of CRC cells, inhibit the development of CRC tumors and enhance the sensitivity of chemotherapy, which is potentially positive for the treatment of CRC (Wang, 2020). It has been reported that Bcl-xL is most frequently amplified in CRC among all cancer types, and the vast majority of CRC exhibits Bcl-xL overexpression and affects CRC survival and progression (Ramesh and Medema, 2020). In addition, the potential prognostic and predictive significance of Bcl2-Associated X (Bax) and Bcl-2 gene expression as well as Bax/Bcl-2 ratio in colorectal cancer has been confirmed (Khodapasand et al., 2015). It has been shown that the nuclear factor kappa-B (NF-κB) signaling pathway plays a key role in apoptosis, tumor promotion, and tumor maintenance, and thus inhibitors of this signaling pathway are useful in CRC treatment (Wang et al., 2009). Notably, p53 shows strong therapeutic potential due to its key function in initiating apoptosis during the cell cycle and in inducing cell arrest and DNA repair in recoverable cells (Yu et al., 2019). In CRC, tumor cell viability, proliferative capacity and angiogenesis can be effectively suppressed by regulating P53 (Malki et al., 2020).

It is not difficult to understand that the pathogenic mechanism of CRC is complex, in addition to the signaling pathways listed above, some other pathogenic mechanisms are also involved, for example, STAT, ROS, β-catenin, AMPK/mTOR, MAPK signaling pathway and so on. The above findings suggest that the occurrence and development of CRC are closely related to apoptosis, and that multiple signaling pathways and targets are involved.

#### 2.1.1 Natural products that regulate apoptosis intervention in CRC

**FIGURE 2:**
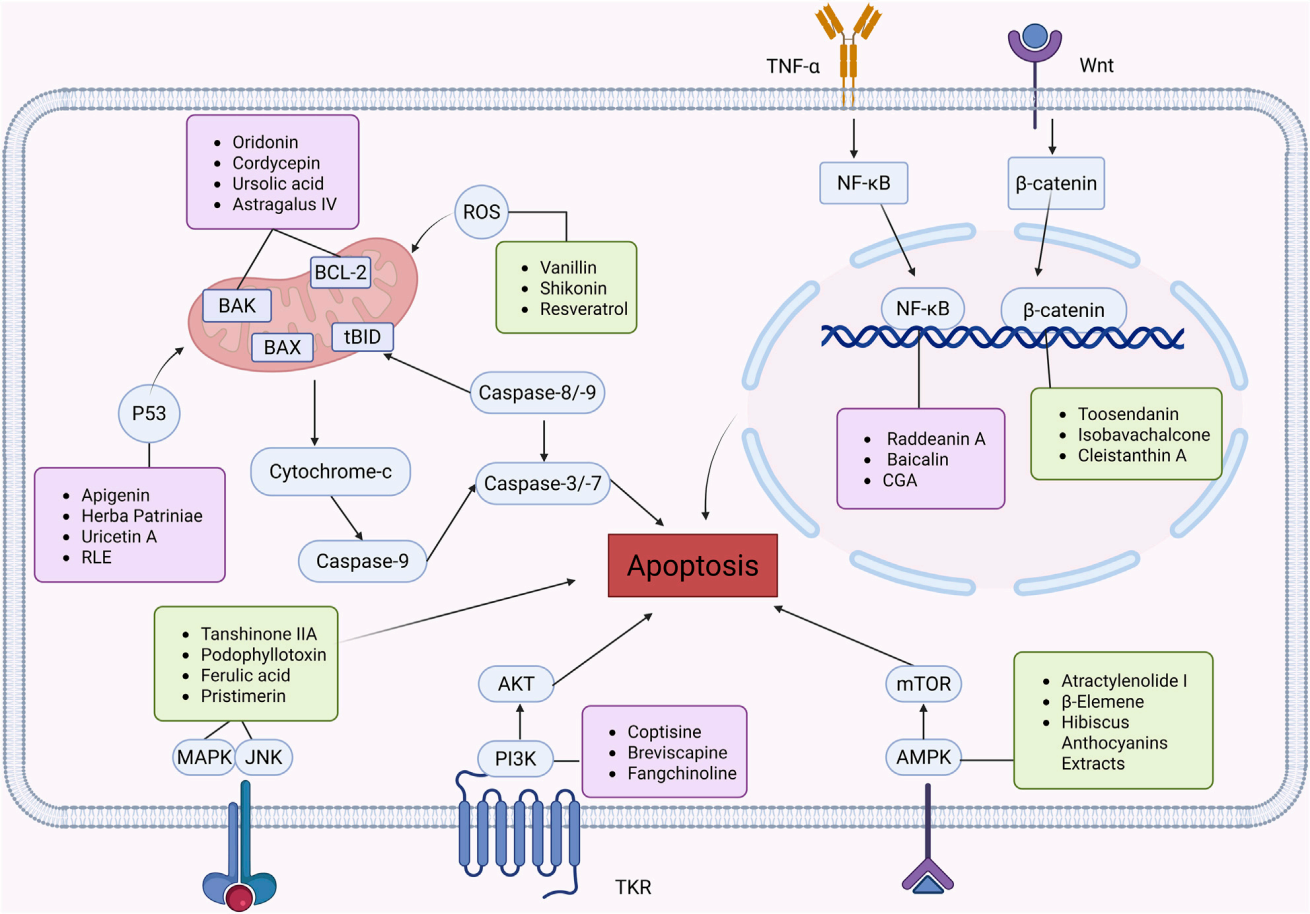
Natural products target ROS, P53, mTOR, NF-κB, β-catenin and other signaling pathways to regulate apoptosis and play a role in intervening CRC. Created with Biorender.com.

Several experimental data have demonstrated that natural products can inhibit the viability and proliferation of CRC cells by inducing the apoptotic pathway, and reduce the weight and volume of tumors in CRC model mice. This therapeutic pathway is associated with ROS, P53, Bax/Bcl-2, STAT, NF-κB, β-catenin, MAPK, AMPK/mTOR, PI3K/AKT, and JNK. This will provide a reliable theoretical basis for natural products to treat CRC (Figure 2).

##### 2.1.1.1 Targeting the ROS signaling pathway

ROS, as an important signaling molecule in redox state, is crucial for the homeostasis of immune cells and is involved in the pathological process of different diseases, and several data have shown that ROS plays an irreplaceable role in inhibiting the progression of CRC by regulating apoptosis (Gao et al., 2023). In response to the above studies, we identified these products that demonstrate the effectiveness of modulating this pathway.

Vanillin is a natural metabolite extracted from *Vanilla planifolia Andrews* and is widely used as a food additive. *In vitro* experiments showed that vanillin significantly inhibited the proliferation of CRC cells and promoted mitochondrial damage and ROS accumulation. *In vivo* experiments in xenograft mice, vanillin (100 mg/kg) combined with 5-FU (30 mg/kg) significantly inhibited tumor growth, upregulated the expression of ROS and caspase-3/9, induced apoptosis of tumor cells, and improved the sensitivity of 5-FU chemotherapy. This may provide a low-toxicity and highly effective drug candidate for the treatment of CRC (Li et al., 2021). However, unfortunately, long-term toxicity assessment, pharmacokinetic studies, and effects on normal tissues have not been addressed, limiting its clinical translational reference value.

Shikonin (SHI) is derived from the naphthoquinones of the dried roots of *Arnebia euchroma (Royle ex Benth.) I.M.Johnst.*, *Lithospermum erythrorhizon Siebold & Zucc.* and *Arnebia guttata Bunge* and was experimentally confirmed to have anti-inflammatory, anti-tumor and promote wound healing properties (Guo et al., 2019). Zhang et al. showed that SHI effectively inhibited the proliferation of HCT116R cells *in vitro* IC_50_, produced a synergistic effect (CI = 0.633) when combined with oxaliplatin (32 μM SHI+32 μM OXA), and significantly promoted ROS generation and induced apoptosis through activation of the endoplasmic reticulum stress pathway (up-regulating p-PERK, p-eIF2α, andATF4) and pro-apoptotic mechanisms (elevating Cleaved Caspase-3, Bax, and decreasing Bcl-2), significantly promoted ROS generation and induced apoptosis. In a CRC mouse model, 4 weeks of treatment with SHI (2 mg/kg) in combination with OXA significantly inhibited tumor growth and improved chemosensitivity without observing significant toxicity (Zhang et al., 2023). However, these experiments did not distinguish whether SHI-induced ROS were target-regulated or chemotoxic products, and therefore complementary mitochondrial function tests (e.g., changes in membrane potential) are recommended to validate the specificity of oxidative stress and avoid false-positive results.

Resveratrol (RSV) is a non-flavonoid polyphenolic compound isolated and obtained from *Reynoutria japonica Houtt.*, a plant of the Polygonaceae family. It has attracted much attention because of its pharmacological properties such as anti-inflammatory, antioxidant, and anticancer (Ren et al., 2021). Fu et al. used CCK8, apoptosis assay, and Western blot in order to explore the anticancer effects and mechanisms of RSV on HCT116 and SW620 cell lines. The results showed that RSV (0, 6, 12 μg/mL) dose-dependently inhibited CRC cell viability and increased apoptosis and ROS levels, while up-regulating the expression of caspase-3/9 and Bax proteins, down-regulating the expression of Bcl-2. The mechanism of action may be that RSV activates the mitochondrial apoptotic pathway by increasing the release of ROS to exert antitumor activity (Fu et al., 2021). This experiment should further improve the results by supplementing the *in vivo* experiments, optimizing the concentration gradient, verifying the ROS dependence and analyzing the cellular heterogeneity, so as to provide a more solid theoretical foundation for the clinical application of RSV.

These studies show a potential role for natural products to induce apoptosis to treat CRC and reduce chemotherapeutic drug resistance, but more clinical and high-quality experimental studies are needed to support this idea.

##### 2.1.1.2 Targeting the P53 signaling pathway

p53 is an important tumor suppressor gene in the human body, playing a key role in biological processes such as cell cycle regulation, DNA repair and apoptosis. Studies have shown that restoration of p53 function or activation of the p53 signaling pathway can promote apoptosis of cancer cells and inhibit tumor growth. It has been noted that it has been widely recognized to ameliorate CRC by modulating the p53 pathway to induce apoptosis (Zhao et al., 2023).

Apigenin is a phytoflavonoid compound found in a wide range of fruits and vegetables such as *Apium graveolens L* which possesses a variety of biological activities, including anti-inflammatory and anticancer properties (Chen et al., 2019). Yang et al. treated HCT116 with apigenin in combination with 5-FU and showed that apigenin significantly increased the Bax/Bcl-2 ratio and upregulated the expression of p53 and ROS in HCT116 and HT29 cells, thereby inhibiting the formation and development of tumor cells. The mechanism may be related to apigenin-mediated activation of the mitochondria-mediated apoptotic pathway by P53, which increases the sensitivity of tumors to 5-FU (Yang et al., 2021). This study was conducted *in vitro* and lacked *in vivo* validation to assess the pharmacokinetics, toxicity and antitumor effects of apigenin. In addition, its unique mechanism or clinical translational value in colorectal cancer was not highlighted.

Li confirmed that Herba Patriniae (HP) and its main component Isovitexin can significantly inhibit the progression of CRC. Transcriptome analysis screened nine active components of HP, among which Isovitexin plays a central role by activating the p53 pathway. In in vitro experiments, HP (40–120 μg/mL) and Isovitexin (5–20 μM) dose-dependently induced apoptosis in HCT116 and SW480 cells and blocked the cell cycle into the G0/G1 phase, with concomitant upregulation of p53 and p21, and downregulation of CDK2. In in vivo experiments using an AOM/DSS-induced CRC model, the HP (1.14–4.56 g/kg) dose significantly inhibited tumor formation while upregulating key molecules of the p53/p21 signaling pathway (Li J. et al., 2023). This study presents only positive results and does not mention the ineffectiveness of other active ingredients or pathways, which may lead to selectivity reporting bias.

Uricetin A (UA) is a natural compound derived from the metabolism of ellagitannins by intestinal bacteria and found in *Berberis vulgaris L.*, which has significant anticancer potential in CRC cells (Totiger et al., 2019). *In vitro* experiments showed that UA inhibited the proliferation of HT29, SW480 and SW620 CRC cell lines in a dose-dependent manner. Treatment with UA (25–100 μM) induced G2/M phase cell cycle arrest, increased pro-apoptotic proteins (p53, p21, cytochrome c, cleaved caspase-3/9), and inhibited anti-apoptotic proteins (Bcl-2, XIAP). *In vivo* studies have emphasized the safety of UA, with no observed genotoxicity and a protective effect against ulcerative colitis (El-wetidy et al., 2021). The present experimental study found that UA induced ROS generation, but did not verify whether ROS is an upstream signal for apoptosis and cell cycle arrest by antioxidant pretreatment experiments, which may confound correlation and causation.

Zong et al. treated HT29 cells with R. molle leaf methanol extract (RLE) (50, 100, and 200 μg/mL) to clarify the effects of RLE on CRC and the potential molecular mechanisms. *In vitro*, RLE significantly inhibited HT29 cell viability and migration in a dose- and time-dependent manner at concentrations of 25–200 μg/mL. Bax and p53 were upregulated and Bcl-2 was downregulated. In addition, RLE treatment caused HT29 cell cycle arrest in S phase, which further inhibited cell proliferation. Gas chromatography-mass spectrometry (GC-MS) analysis showed that RLE contained 17 potential anticancer components, which may be the material basis of its anticancer activity (Zong et al., 2021). However, there are some limitations of this study. The study was conducted only *in vitro* and lacked *in vivo* experimental validation to assess the efficacy and safety of RLE *in vivo*. In addition, although gas chromatography-mass spectrometry (GC-MS) analysis identified a variety of potential anticancer components, it did not further identify which components were the main active substances or explore the synergistic effects among them.

In addition, we have identified a number of apoptosis-inducing products (Table 1), which can also play an important role in intervening in the course of CRC. All of these studies suggest that these products are able to induce apoptosis in CRC cells through P53 signaling as an effective pathway.

**TABLE 1:** Natural compounds target apoptosis signaling pathway to treat CRC.

Extract	Origination	Structure	Models	Biological effects	Results	References
vanillin	*Vanilla planifolia*	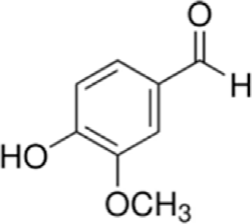	SW480/HT-29 Cells CRC model mice	ROS↑Caspase-3/9↑	Promote mitochondrial damage Induce apoptosis Inhibit CRC tumor growth	Li et al. (2021)
Shikonin	*Arnebia euchroma (Royle) Johnst*	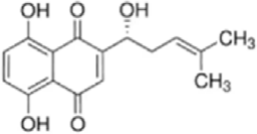	HCT116 Cells CRC model mice	ROS↑ Caspase3↑Bax↑ Bcl-2↓	Inhibit CRC tumor growth Reduce OXA resistance	Zhang et al. (2023)
Resveratrol	*Polygonum cuspidatum*	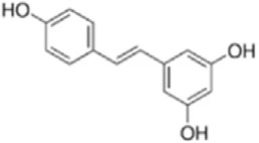	HCT116/SW620 Cells	ROS↑Caspase3↑Bax↑ Bcl-2↓	Inhibit CRC cell viability	Fu et al. (2021)
Apigenin	*Apium graveolens* L	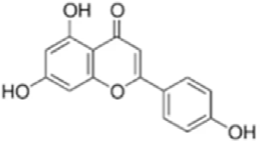	HCT116/HT29 Cells	Bax/Bcl-2↑ P53↑ ROS↑	Reduce 5-FU resistance Inhibit viability and proliferative capacity	Yang et al. (2021)
Herba patriniae	*Thlaspi arvense L*	------	HCT116/SW480 Cells CRC model mice	P53↑	Inhibit tumor formation Induce apoptosis G0/GI cell cycle arrest	Li J. et al. (2023)
Urolithin A	*Punica granatum L.*	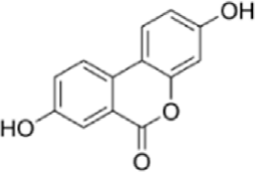	HT29/SW480/SW620 Cells	caspase-3/9↑p53↑Bax↑ ROS↑Bcl-2↓	Reduce the cellular activity of CRC G2/M cell cycle arrest Inhibit tumor cell production	El-Wetidy et al. (2021)
Piper nigrum Extract	*Piper nigrum*	------	HT29 Cells CRC model mice	caspase-3↑p53↑Bax↑Bcl-2↓	Induce apoptosis Inhibit tumor growth	Wu et al. (2023)
Ethanol extract of Spica Prunellae	*Spica Prunellae*	------	HT29 Cells CRC model mice	STAT3↓ Bax/Bcl-2↑	Inhibits cell viability and tumor cell angiogenesis	Lin et al. (2013)
Convallatoxin	*Convallaria majalis L.*	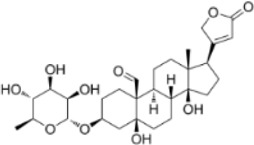	HCT116 Cells CRC model mice	STAT↓ Bcl-2↑	Reduce cell viability Inhibit tumor cell proliferation, migration and angiogenesis	Zhang et al. (2020)
Oridonin	*Rabdosia rubescens*	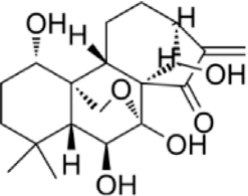	HCT116/LoVo Cells CRC model mice	Caspase-3/9↑Bax↑ Bcl-2↓	Inhibit tumor growth Reduce tumor volume	Yang et al. (2021)
Ursolic acid	*Ligustrum lucidum W.T.Aiton*	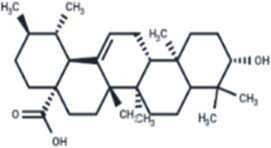	RKO Cells	Caspase-3/8/9↑Bax↑ROS↑ Bcl-2↓	G0/G1 cell cycle arrest Inhibit proliferation and growth	Zheng et al. (2021)
Astragaloside IV	*Var. mongholicus (Bge.) Hsiao*	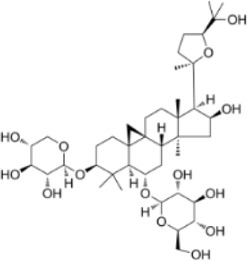	HT29/SW480 Cells CRC model mic	Caspase-3/7/9↑Bax↑ Bcl-2↓	Inhibits the proliferation Suppress tumor growth G0 cell cycle arrest	Sun et al. (2019)
Raddeanin A	*Anemone raddeana Regel*	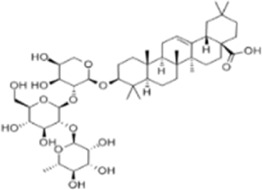	SW480/Caco-2/HT-29/LOVO Cells CRC model mice	Caspase-3/9↑Bax↑Bcl-2↓ NF-κB↓	Inhibit cell proliferation Suppress colon cancer cell growth, mitochondrial dysfunction and cell cycle arrest	Wang et al. (2018)
Chlorogenic acid	*Lonicera japonica Thunb*	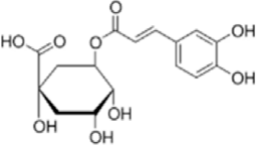	HT-29/HEK-293 Cells	Caspase-3/9↑Bax↑ROS↑Bcl-2↓ NF-κB↓	Reduced CRC cell line viability and proliferation and cell cycle arrest	Ranjbary et al. (2023)
Baicalin	*Scutellaria baicalensis*	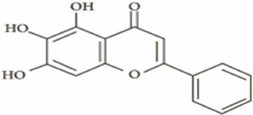	HCT-116/CT26 Cells CRC model mice	TLR4↓NF-κB p65↓p-IκBα↓ Bax/Bcl-2↑	Inhibit CRC cell migration and invasion Improve the tumor immunosuppressive environment Improve tumor immunocompetence	Song et al. (2023)
Toosendanin	*NeemMeLia toosendanSieb.et Zucc*	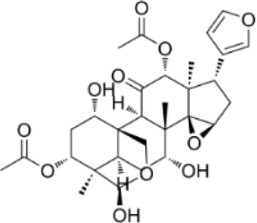	SW480 Cells CRC model mice	Caspase-3/9↑Bax↑β-catenin↓	Inhibit tumor volume and weight Reduce tumor growth rate	Wang et al. (2015)
Isobavachalcone	*Psoralea corylifolia Linn*	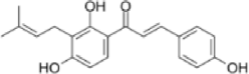	HCT116/SW480 Cells	Caspase-3/9↑Bax↑β-catenin↓	Inhibit proliferation and colony formation	Li et al. (2019)
Atractylenolide I	*Atractylodes macrocephala Koidz*	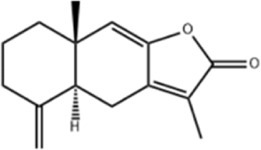	COLO20/HCT116 CRC Cells CRC model mice	Caspase-3↑Bax↑ Bcl-2↓AMPK/mTOR↓	Inhibit tumor cell growth.	Wang et al. (2020)
β-Elemene	*Curcuma longa.L*	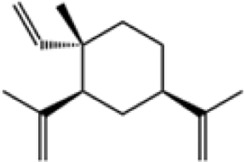	HT29 Cells CRC model mice	caspase-3/9↑ Bax↑ ROS↑ Bcl-2↓	Reduce tumor volume and weight Induce apoptosis Inhibit tumor progression.	Wang et al. (2022)
Tanshinone IIA	*Salvia miltiorrhiza Bunge*	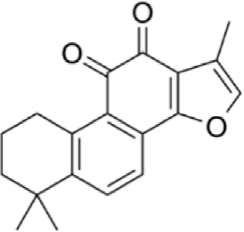	SW620 Cells CRC model mice	p38MAPK↑	Inhibited tumor growth and CRC cell viability and proliferation	Zhang et al. (2023)
Ferulic Acid	*Rhizoma Ligustici*	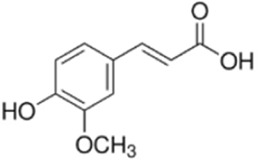	CT26 Cells CRC model mice	MAPK↑Caspase-3/9↑Bax↑Bcl-2↓	Alleviate tumor-induced weight loss Inhibit tumor growth	Chen et al. (2023)
Pristimerin	*Melicope madagascariensis (Baker) T.G.Hartley*	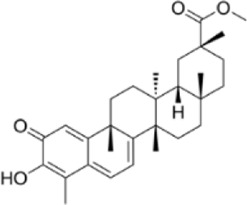	HCT116/SW620 Cells CRC model mice	ROS↑Caspase-3↑JNK↑	Inhibit tumor cell growth and cell activity	Zhao et al. (2023)
Garcinone E	*Garcinia mangostana L*	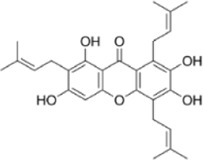	HT29/Caco-2 Cells CRC model mice	Caspase-3/9↑Bax↑Bcl-2↓ROS↑ JNK↑	Inhibit tumor growth rate Increase the area of tumor tissue necrosis	Li L. et al. (2023)
Isolinderalactone	*Lindera aggregata (Sims) Kosterm*	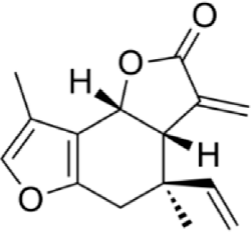	HCT116/HT29 Cells	Caspase-3/9↑JNK↑	Inhibit cell proliferation and colony formation Induce apoptosis Induce G2/M cell cycle arrest	Kwak et al. (2022)
Chaetocin	*Trichoderma spp. fungi*	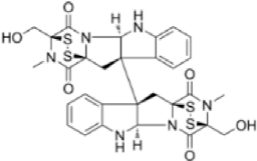	HCT116 Cells CRC model mice	Caspase-3/9↑Bax↑Bcl-2↓ ROS↑ JNK↑	Inhibit cell growth and cell viability Promote G2/M-phase blockade	Wang et al. (2021)
Coptisine	*Coptis chinensis Franch*	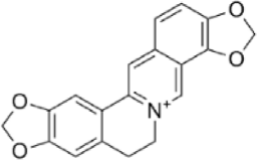	HCT116 Cells CRC model mice	Bcl-2↓P13K/AKT↓Caspase-3↑	Inhibit PI3K/AKT Induce mitochondria-mediated apoptosis	Han et al. (2018)
Breviscapine	*Erigeron breviscapus*	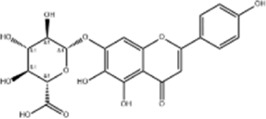	HCT116/SW480 Cells CRC model mice	Caspase-3↑ p53↑ P13K/AKT↓	Suppression of tumor weight and volume Inhibition of CRC cell activity and proliferative capacity	Niu et al. (2023)
Fangchinoline	*Stephania tetrandra*	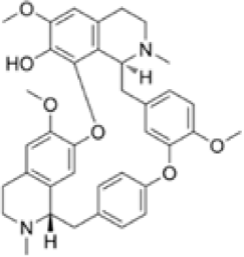	DLD-1/LoVo Cells CRC model mice	Bax↑Bcl-2↓P13K/AKT↓	Induce apoptosis G1 cell cycle arrest	Jiang et al. (2021)
Dieckol	*Lantana camara L.*	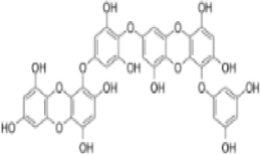	HCT116 Cells	Bax↑caspase-3.9↑Bax↑Bcl-2↓ P13K/AKT↓	Inhibit CRC cell viability and proliferation	Dai et al. (2023)
procyanidin B2	*Vitis vinifera L.*	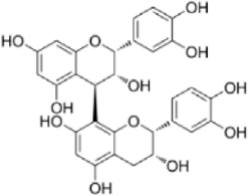	HT29/LoVo Cells CRC model mice	Beclin1↑LC3 II↑Atg5↑ caspase-3↑ Bax↑Bcl-2↓	Inhibit CRC cell viability and proliferative capacity Reduce tumor cell weight	Zhang et al. (2019)

##### 2.1.1.3 Targeting the STAT signaling pathway

Increasing evidence suggests that STAT-related pathways play an important role in human disease pathology and pharmacological mechanisms. Some studies have found that apoptosis can be induced by regulating the STAT signaling pathway to inhibit tumor angiogenesis, which brings new hope for the treatment of CRC (Ghasemian et al., 2023).

Spica Prunellae is the fruiting spike of the plant drug *Prunella vulgaris L*. distributed in Northeast Asia, which has significant anticancer activity (Kim et al., 2020). Lin et al. intervened a CRC mouse xenograft model with ethanol extract of Erythrina spicata (EESP) (6 g/kg) for 16 days. *In vivo* experiments showed that EESP significantly inhibited the growth of transplanted tumors in CRC nude mice. In in vitro experiments, EESP inhibited HT29 cell viability in a dose-dependent manner, with 63% inhibition by 2 mg/mL treatment for 24 h. Mechanistic studies have shown that EESP induces apoptosis and inhibits cell proliferation and angiogenesis through inhibition of STAT3 phosphorylation, upregulation of the pro-apoptotic protein Bax/Bcl-2 ratio, downregulation of the cell cycle proteins Cyclin D1 and CDK4, and reduction of VEGF-A and VEGFR-2 (Lin et al., 2013). The specific composition and content of EESP were not clarified in this experiment, and it should be necessary to analyze the fingerprints by techniques such as LC-MS/MS and verify the synergistic effect of a single component or combination. The current experiment could not confirm the main active substances.

Convallatoxin a natural triterpenoid derived from *Adonis amurensis Regel & Radde*, has significant anticancer activity (Liu J. et al., 2022). *In vitro* experiments showed that Convastatin (0–100 nM) significantly downregulated STAT3 phosphorylation levels by inhibiting the interaction of JAK2/STAT3 and mTOR/STAT3 signaling pathways, thereby inhibiting the proliferation, migration and invasion of CRC cells and inducing apoptosis. In in vivo experiments, oral administration of combretastatin at doses of 50 and 150 μg/kg significantly inhibited the growth of HCT116 cell xenograft tumors with low toxicity to normal cells (Zhang et al., 2020). However, the experiment lacked rigor; 30 nM CNT was non-toxic to HUVEC cells in the *in vitro* assay, but the dose used in the *in vivo* assay was 150 μg/kg which raises questions about the appropriateness of the concentration used.

In conclusion, these natural products may be a new way to treat CRC by modulating the STAT signaling pathway. However, it is regrettable that there are fewer such products, and in the future, we should make more efforts to explore it.

##### 2.1.1.4 Targeting the Bax/Bcl-2 signaling pathway

Bax and Bcl-2 are the main members of the Bcl-2 family. Bax promotes cell death by permeabilizing the outer mitochondrial membrane in response to different cellular stresses. In contrast, Bcl-2 prevents apoptosis by inhibiting Bax activity. Furthermore, it is exciting to note that modulation of the Bax/Bcl-2 ratio has potentially positive implications for CRC intervention. The results of some studies have shown that this class of natural products can effectively inhibit CRC cell viability and proliferation by down-regulating the anti-apoptotic protein Bcl-2.

Oridonin (Ori) is a diterpenoid isolated from the plant drug *Isodon rubescens (Hemsl.) H. Hara.* Modern pharmacological studies have shown that Ori has anti-inflammatory and anticancer activities (Liu X. et al., 2020). *In vitro* experiments showed that Ori inhibited the proliferation of HCT-116, LoVo and other CRC cells and induced apoptosis in a concentration-dependent manner (IC_50_ values of 10–20 μmol/L). The mechanism of action includes activation of the mitochondrial apoptotic pathway, upregulation of Bim/Bax, downregulation of Bcl-2, and activation of caspase-9/3. *In vivo* experiments demonstrated that intraperitoneal injection of Ori (10 mg/kg) markedly inhibited the growth of HCT116 and LoVo xenograft tumors and synergistically potentiated the effects of chemotherapeutic agents on drug-resistant CRC cells (Yang et al., 2015). In this study, only two high miR-32 expressing cell lines, HCT-116 and LoVo, were selected as the main subjects, and other low expressing cell lines were not included for comparative validation, which may lead to one-sided conclusions.

Cordycepin is a metabolite extracted from *Ophiocordyceps sinensis (Berk.) G.H.*, which has outstanding effects in anti-inflammatory and anti-tumor effects (Deng et al., 2022). Li et al. treated HCT116 cells with Cordycepin (0, 62.5, 135, 270, and 540 μM) for 24 h. The results showed that cordycepin induced cell death through Bax-dependent mitochondrial apoptotic pathway with a half inhibitory concentration (IC_50_) of 434 μM. In addition, Cordycepin can induce G1/S phase cell cycle arrest through upregulation of the p53/p21 pathway (Li et al., 2019). However, the study has not yet involved *in vivo* experimental validation and pharmacokinetic analysis, and its efficacy and safety in animal models need to be further explored.

Ursolic acid (UA) is a natural triterpenoid extracted from the dried root of Chinese kiwifruit *Actinidia chinensis Planch*, a plant in the kiwifruit family, and has significant anticancer activity (Zhao et al., 2023). Zheng et al. reported that UA inhibited the growth of CRC cells through a multi-target regulatory mechanism, significantly induced apoptosis in RKO cells, blocked the cell cycle in G0/G1 phase, and downregulated the expression of Bcl-2, upregulated the expression of Bax, and activated the caspase-3/8/9 cascade response. In addition, UA can inhibit EMT by up-regulating the epithelial marker E-Cadherin and down-regulating the mesenchymal marker N-Cadherin, thereby inhibiting tumor metastasis. This *in vitro* study demonstrated that UA has significant anti-tumor effects, which provides an important basis for subsequent *in vivo* studies. However, this study did not involve *in vivo* validation and pharmacokinetic analysis, and further studies in animal models are needed to evaluate its efficacy and safety *in vivo* (Zheng et al., 2021).

Astragalus IV (AS-IV) was extracted from the roots of *Astragalus mongholicus Bunge*, showing potent biological activities in anti-inflammatory, antioxidant, and anticancer properties (Zhang et al., 2020). Sun et al. treated CRC xenograft mice by AS-IV (20 mg/kg) for 30 days. The results showed that AS-IV dose-dependently inhibited the proliferation of CRC cells and suppressed tumor growth, activated the levels of Caspase-3/7/9, Bax, inhibited the expression of Bcl-2, and promoted the cell cycle arrest at G0 phase (Sun et al., 2019). However, the concentration gradient and positive control group were not set in this experiment, which needs to be further improved in the future.

The above findings suggest that all these products are able to modulate endogenous mitochondrial pathways such as the anti-apoptotic protein Bcl-2 in order to achieve the treatment of CRC, and such drugs may be potential candidates for the treatment of CRC in the future.

##### 2.1.1.5 Targeting the NF-κB signaling pathway

NF-κB is a multifunctional key transcriptional regulator involved in various biological processes, and plays an important role in the process of disease occurrence and development. Therefore, the use of natural products to intervene in NF-κB has become an important measure for the treatment of diseases.

It has been reported that some natural products can mediate NF-κB to regulate apoptosis, which can effectively inhibit CRC progression. For example, Raddeanin A (RA), a triterpenoid isolated from *Anemone raddeana Regel*, has anti-inflammatory, anticancer, and angiogenesis inhibitory properties (Naz et al., 2020). Wang et al. treated a CRC xenograft mouse model with RA (1.25, 2.5, 5.0 mg/kg) and adriamycin (5 mg/kg) as a positive control for 14 days. The experimental results showed that intraperitoneal injection of RA at doses of 2.5–5.0 mg/kg significantly inhibited the growth of SW480 xenograft tumors. Mechanistic studies showed that RA downregulated the levels of p-LRP6, p-AKT and intranuclear β-catenin and blocked the expression of c-Myc and Cyclin D1. In addition, *in vitro* experiments showed that RA inhibited the proliferation of SW480, LOVO and other CRC cells in a dose-dependent manner, with IC_50_ of 1.81–9.65  μM at 24 h (Wang et al., 2018). This provides an important basis for the development of novel CRC therapeutic strategies.

In addition, there are several apoptosis-inducing products (Table 1), which can likewise play an important role in intervening in the course of CRC. The above studies suggest that the NF-κB-related signaling pathway is a meaningful pathway for natural products for CRC therapy.

##### 2.1.1.6 Targeting the β-catenin signaling pathway

β-catenin signaling is the most critical pathway regulating cell invasion, migration, proliferation and differentiation, and its important role in tumor diseases has been well described. Some studies have shown that β-catenin is closely associated with CRC progression. Our discovery of several natural products mediating the β-catenin pathway for the treatment of CRC is of great significance.

Toosendanin (TSN) is a triterpenoid extracted from the dried ripe fruits of the neem plant drug *Melia azedarach L.* and possesses antioxidant, anti-inflammatory, and anticancer pharmacological activities (Li et al., 2024). Wang et al. demonstrated the efficacy of TSN *in vivo* in SW480 xenografted nude mice by injecting TSN (0.15, 0.3 mg/kg) for 14 consecutive days in the absence of a positive control group, with daily intraperitoneal injections of 0.15/0.30 mg/kg significantly inhibiting tumor growth (p < 0.01), and reductions in tumor weight and volume correlating with reduced nuclear localization and upregulation of GSK-3β expression in β-catenin. Catenin nuclear localization and upregulation of GSK-3β expression. *In vitro* studies using SW480 cells demonstrated that TSN (0.125–0.5 μM) inhibited cell proliferation and S-phase cell cycle arrest in a dose- and time-dependent manner (Wang et al., 2015). However, there was no significant difference in tumor volume between the high-dose TSN (0.3 mg/kg) and low-dose (0.15 mg/kg) groups in the *in vivo* experiments, suggesting that there may be a dose saturation effect or experimental design limitations. Further validation of the dose range or extension of the administration time is needed to confirm the efficacy.

Cleistanthin A (CA) is a natural compound extracted from *Phyllanthus taxodiifolius Beille* with significant anticancer activity (Pradheepkumar and Shanmugam, 1999). Jearawuttanakul et al. intervened HCT116 and SW480 cells using CA (1 and 10 μM). The experimental results confirmed that CA induced apoptosis in CRC cells, inhibited cell proliferation and viability, and suppressed their invasion and migration ability. Mechanistic studies showed that CA induced apoptosis by inhibiting the Wnt/β-catenin signaling pathway, down-regulating the expression of Survivin and target genes CCND1 and AXIN2, and inhibiting the nuclear translocation and transcriptional activity of β-catenin. In addition, CA significantly inhibited cell migration and invasion within 24 h, and its effects were associated with inhibition of V-type ATPase activity and focal adhesion kinase (FAK) phosphorylation (Jearawuttanakul et al., 2020). The limitations of this study are the lack of *in vivo* experiments to validate the efficacy and safety of CA, the potential toxicity to normal tissues and the tolerability of long-term treatment have not been clarified and further studies are needed.

In addition, there are several apoptosis-inducing products (Table 1) that can also function by intervening in the course of CRC. The above studies suggest that modulation of the β-catenin signaling pathway may be a new avenue for the treatment of CRC.

##### 2.1.1.7 Targeting the AMPK/mTOR signaling pathway

Atractylenolide I (ALT-1), a natural sesquiterpene lactone from the dried rhizomes of *Atractylodes macrocephala Koidz.* in the Asteraceae family, is known for its antioxidant, anti-apoptotic, and anti-tumor properties (Liu et al., 2021). Wang et al. intervened CRC xenograft model mice by ALT-1 (25 and 75 mg/kg), administered intraperitoneally for 22 days without a positive control group. ALT-1 significantly upregulated the levels of Bax, Caspase-3 and downregulated the expression of Bcl-2 in a dose-dependent manner, reduced the size and weight of tumors, and inhibited aerobic glycolysis and AKT/mTOR activation, and the effect of 75 mg/kg ALT-1 was more obvious. In addition, *in vitro* experiments also showed that ALT-1 (0, 80, 150, 200 μM) reduced the viability and proliferation of COLO205 and HCT116 cell lines, and downregulated the phosphorylation of proteins associated with the AKT/mTOR pathway to inhibit the growth of tumor cells (Wang et al., 2020). This study still has some shortcomings, such as the stability and safety of ATL-1 has not been evaluated in this experimental ATL-1. In addition, extensive clinical studies should be conducted to determine its specific efficacy.

β-Elemene, a sesquiterpene compound extracted from the dried rhizome of the ginger plant drug *Curcum*a longa.L., has shown potent antitumor effects (Feng et al., 2024). *In vitro* experiments, treatment of human CRC cell lines DLD-1 and HT29 with β-Elemene at concentrations ranging from 25 to 600 μmol/L significantly decreased cell viability and induced G2/M phase block. Mechanistic studies showed that β-Elemene activated AMPK phosphorylation by increasing intracellular ROS levels, which in turn inhibited mTOR signaling. In in vivo experiments, intraperitoneal injection of β-Elemene into hormonal nude mice at doses of 25 mg/kg and 50 mg/kg significantly inhibited the growth of HT29 graft tumors (Wang et al., 2022). The present study proposes the ROS/AMPK/mTOR pathway as the central mechanism, but does not exclude the interference of other potential pathways (e.g., PI3K/Akt). For example, whether β-elemene directly regulates AMPK phosphorylation or whether other upstream kinases are involved needs to be further verified by gene silencing or drug intervention experiments.

##### 2.1.1.8 Targeting the MAPK signaling pathway

The MAPK cascade response is a key pathway that regulates a variety of cellular processes such as cell growth, proliferation, differentiation, migration, and apoptosis, and plays a critical role in the outcome and sensitivity of anticancer therapy. Therefore, intervention of MAPK using natural products has emerged as a potential means of treating diseases.

Podophyllotoxin (PT), a lignan compound from the roots and rhizomes of *Oncorhynchus mykiss*, possesses significant anticancer activity (Motyka et al., 2023). Lee et al. reported that PT (0, 0.1, 0.2, and 0.3 μM) inhibited the proliferation of HCT116 CRC cells in a concentration-dependent manner and activated the p38 MAPK signaling pathway by inducing ROS production, which led to cell cycle arrest in G2/M phase and triggered apoptosis (Lee et al., 2021). However, there are some limitations in this study, such as no *in vivo* experiments were performed to validate the antitumor effect and safety of PT.

Ferulic acid (FA) is a phenolic compound present in plant medicines such as *Angelica sinensis (Oliv.) Diels* and *Conioselinum anthriscoides (H.Boissieu) Pimenov & Kljuykov*, which exhibits antioxidant, anti-oxygen radical, and inhibitory properties against tumor development (Li et al., 2021). Chen et al. intervened CT26 cells with FA (0–400 μM), which inhibited the viability and proliferation ability of CT26 cells, and TUNEL staining showed that FA effectively promoted the overall apoptosis of cancer cells *in vivo*. *In vivo* experiments further showed that FA (20, 40, and 80 mg/mL/kg) gavaged BALB/c tumor-bearing mice, and the 80 mg/kg group showed a 41% reduction in tumor volume and a 37% reduction in weight, with no impairment of liver and kidney functions. Mechanistic studies have found that FA downregulates BCL-2 and upregulates BAX through activation of JNK/ERK phosphorylation, while promoting LC3-II expression (Chen et al., 2023). However, this study did not explore the synergistic effect of FA with clinical chemotherapeutic agents. In the future, a large number of clinical trials should be conducted to clarify its true clinical efficacy and synergistic effects.

All of the above studies suggest that such products can inhibit CRC progression through MAPK signaling as an effective pathway.

##### 2.1.1.9 Targeting the JNK signaling pathway

The JNK signaling pathway is a conserved response to multiple internal and external cellular stress signals. In addition, this signaling pathway not only maintains the regulation of apoptosis, growth, proliferation, differentiation, migration, and invasion, but is also a key player in regeneration, tumorigenesis, and other pathologies in a variety of diseases. Therefore, targeting these products to modulate the JNK pathway for the treatment of CRC is of positive significance.

Pristimerin is a natural product of quinone methylated triterpenoids present in *Celastrus paniculatus Willd.*, which has been shown potent anti-cancer effects (Sun et al., 2022). Zhao et al. investigated the mechanism of action of Pristimerin in the treatment of CRC mice induced by injection of HCT116 cells. *In vivo* experiments showed that Pristimerin (0.125–0.5 mg/kg) significantly inhibited the growth of HCT116 xenograft tumors and the formation of intestinal polyps in APC mice without significant toxicity. The compound induced the expression of the pro-apoptotic protein Noxa through activation of the ROS/ER/JNK pathway. In addition, this experiment was further validated in HCT116 and SW620 cell models, which showed that Pristimerin inhibited cell proliferation and induced apoptosis in a dose-dependent manner (0.4–0.8 μM), thus exerting anti-tumor effects (Zhao et al., 2021). In this study, the *in vivo* experimental Pristimerin dose was significantly lower than the positive control docetaxel, but the inhibition rate was similar (62.59% vs 60.19%), and there may be an inadequate explanation of the dose-effect relationship.

Garcinone E (GAR E) is a xanthone compound extracted from *Garcinia mangostana L.*, which possesses biological properties such as anti-inflammatory, antioxidant, and anticancer (Mohamed et al., 2022). Li et al. used HT29 and Caco-2 CRC cells as *in vitro* models. The results showed that GAR E activated the JNK signaling pathway by inducing ROS accumulation, which triggered mitochondrial dysfunction and apoptosis, and blocked the cell cycle into the Sub G1 phase. The specific manifestations of GAR E are elevated Bax/Bcl-2 ratio, activation of PARP and caspase 3/9, and significant increase in JNK1/2 phosphorylation. *In vivo* studies have further demonstrated that 2.5–5 mg/kg of GAR E significantly inhibited the growth of HT29 xenograft tumors, induced the expression of apoptosis-associated proteins in tumor tissues, and reduced the area of tumor necrosis without causing significant toxicity (Li L. et al., 2023). However, this study still has some limitations, and the *in vivo* experiments only used a single cell line model, which needs to be extended to more CRC subtypes for validation.

Isolinderalactone (ILL) is a bioactive sesquiterpene lactone derived from *Lindera aggregata (Sims) Kosterm.* with antitumor properties (Hwang et al., 2019). It was reported that Kwak et al. treated HCT116 and HT29 cell lines with ILL to elucidate the mechanism of action of ILL. The results showed that ILL (3–9 μM) dose-dependently inhibited the proliferation and colony formation of OXA-sensitive and CRC cells through activation of JNK/p38 MAPK signaling, and induced G2/M phase arrest, ROS accumulation, and apoptosis. *In vivo* validation using a xenograft model further supports the tumor suppressive activity of ILL, although specific dosage details were not explicitly provided (Kwak et al., 2022). Limitations of this study include the lack of in-depth *in vivo* toxicity analysis and incomplete exploration of chemotherapy combinations.

As described above, TanIIA has potent antitumor activity. Qian et al. experimentally verified the antitumor pharmacological effects of TanIIA (20 mg/kg) by CRC xenograft model mice treated by gavage for 10 days. Tan IIA can mediate the translocation of Bax to the mitochondria, which can lead to mitochondrial damage, activation of Caspse-3/9, ROS levels ultimately leading to mitochondria-dependent apoptosis and inhibiting tumor cell progression. In addition, in SW480 cell assay, CK-8 and colony formation assay showed that cell proliferation and viability were inhibited, flow cytometric analysis showed that Tan IIA induced apoptosis in CRC cells, and western blot analysis showed that the expression of Bax protein was significantly upregulated in Tan IIA group, and the expression of Bcl-2 protein was significantly reduced. Although this experiment verified the inhibitory effect of Tan IIA on CRC cells and xenografted CRC mice (Qian et al., 2023). However, compounds that are structurally similar but do not contain a quinone molecule (e.g., Tanshinone I) were not used as negative controls in this experiment, making it difficult to rule out nonspecific toxicity or PAINS effects. For example, the quinone structure may induce oxidative stress directly rather than through specific signaling pathways.

Chaetocin is a natural metabolite isolated from *Trichoderma spp. fungi* belonging to the genus Thioketopyrazine, and it possesses a variety of bioactivities and pharmacological functions, such as anticancer, inhibition of cell proliferation or angiogenesis, etc., (Jiang et al., 2021). Wang et al. used Chaetocin (0.5 mg/kg) to treat CRC xenograft model mice for 18 days. The results showed that Chaetocin upregulated the levels of Bax, Caspase-3/9, ROS, JNK and downregulated the expression of Bcl-2 to promote apoptosis to inhibit the tumor volume and weight and to improve the sensitivity of 5-FU drug. Notably, Chaetocin (0–2 μM) was able to inhibit cell growth and cell viability and promote G2/M-phase blockade in in vitro experiments, exerting anticancer effects (Wang et al., 2021). Indicators of drug toxicity (e.g., weight change, liver and kidney function) were not reported in this study to assess the safety of Chaetocin, which is critical for clinical translation.

##### 2.1.1.10 Targeting the PI3K/AKT signaling pathway

The PI3K/AKT pathway is a key signaling cascade involved in the regulation of cell growth, survival, and metabolism, and is also considered one of the most altered molecular pathways in malignant tumors. Therefore, modulation of the PI3K/AKT signaling pathway by these several products for the treatment of CRC might be a new avenue.

Coptisine (COP), an isoquinoline alkaloid in the dried rhizomes of the buttercup plant drug *Coptis chinensis Franch.,* has been shown to possess pharmacological properties such as anti-inflammatory, antioxidant, and anticancer (Lu et al., 2023). Han et al. elucidated the anti-tumor mechanism of action of COP by HCT116 xenografts in CRC model mice without a positive control group. The results showed that COP (50, 100, 150 mg/kg) was able to alleviate tumor growth and tumor weight in CRC model mice. When the COP dose was 150 mg/kg, the effect was more significant, and COP also decreased the levels of Bcl-2, P13K/AKT and upregulated the expression of Caspase-3. The mechanism is related to the inhibition of PI3K/AKT and activation of mitochondria-mediated apoptosis pathway to exert anti-tumor effects. In addition, through experiments, we also found that COP significantly inhibited the viability, proliferation and cell migration of HCT-116 cell line (Han et al., 2018). However, no study related to pharmacological toxicity and clinical trials was conducted in this experiment, and the safety and clinical therapeutic effects are worrisome.

Breviscapine is a flavonoid glycoside extracted from the plant drug *Erigeron breviscapus (Vaniot) Hand. -Mazz.*, and it has various pharmacological applications, such as anti-inflammatory and antitumor activities (Wen et al., 2021). The effects and mechanisms of Breviscapine in CRC were clarified experimentally by Niu et al. Breviscapine (0, 12.5, 25, 50, 100, and 200 μM) was able to effectively inhibit the viability and proliferation of HCT116 and SW480 cell lines in a dose-dependent manner, and limit the migration and invasion of CRC cells. *In vivo* experiments further showed that Breviscapine (40 mg/kg) was able to inhibit tumor volume and weight, while 5-FU (25 mg/kg) also showed this therapeutic effect and had a synergistic effect with Breviscapine (Niu et al., 2023). However, key factors such as angiogenesis and immune microenvironment were not addressed in this discussion. These factors may interact with the PI3K/AKT pathway, and further studies are needed to fully reveal the antitumor mechanism of Breviscapine.

Fangchinoline (FAN) is an alkaloid extracted from *Stephania tetrandra S. Moore*, which possesses a variety of biological and pharmacological activities such as anti-inflammatory and anti-tumor (Mérarchi et al., 2018). Jiang et al. experimentally explored the mechanism of action of FAN on CRC. *In vivo* and *ex vivo* experiments were used to evaluate its effects. Using MTT, wound healing assay, Transwell and Western blot assays, it was found that FAN (0–9 μM) inhibited cell migration and invasion, suppressed the expression of Caspase-3/9 and Bax, downregulated the level of Bcl-2, and induced apoptosis and G1-phase cell cycle arrest to reverse CRC progression. Moreover, the therapeutic effect of FAN (0.1 and 0.5 mg/mL) was also demonstrated in an *in vivo* CRC xenograft model mouse, and effectively suppressed the tumor volume and weight, and this experiment elucidated that FAN blocked the PI3K/AKT signaling pathway in order to exert the anti-tumor activity (Jiang et al., 2021). In this study, the FAN dose used for the *in vivo* experiments was not converted to a human equivalent dose. The difference between this dose and clinically used chemotherapeutic agents (e.g., oxaliplatin) is significant, and the safety window needs to be determined by toxicology studies and pharmacokinetic analysis.

Table 1 lists other natural products that inhibit CRC progression by modulating apoptosis induced by the PI3K/AKT-related signaling pathway. The above studies suggest that modulation of PI3K/AKT signaling by these products is considered an important alternative to inhibit CRC progression and cell cycle arrest.

### 2.2 Autophagy

Cellular autophagy refers to the transportation of damaged, denatured or senescent proteins and organelles in the cell to the lysosome, which digests and degrades them (Liu et al., 2023). Meanwhile, the energy and small molecules produced by degradation, such as amino acids, are available for reuse by the cell, which is important for the cell to maintain homeostasis of the organism. Cellular autophagy is not only a self-protection mechanism of cells, but also closely related to a variety of physiological and pathological processes. The nature of autophagy can be summarized as intracellular membrane rearrangement, and the process of its occurrence can be divided into four stages: ① the initiation of autophagy: the formation of autophagosome initiation in the cytoplasm, called phagocytosis vesicles; ② the formation of autophagosomes: autophagosomes, through the extension and expansion, ultimately form the double-layer membrane structure wrapped around the cytoplasm and the cell organelles; ③ autophagosomes fused with lysosomes: autophagosomes and lysosomes fused to form autophagolysosomes, which contain a variety of hydrolytic enzymes that can degrade its contents; ④ fusion of autophagosomes: the fusion of autophagosomes with lysosomes is a sign that autophagy is effective, and the degraded products will be released for reuse by the cell (Cao et al., 2021). Several data have shown that cellular autophagy plays an important role in tumor formation and development. Therefore, targeting and regulating cellular autophagy to inhibit tumor formation is considered a promising strategy (Debnath et al., 2023).

The occurrence of cellular autophagy is the joint participation of multiple signaling molecules, which affects the regression and prognosis of the disease. Ma et al. showed that cellular autophagy was regulated by inhibiting the PI3K/AKT/mTOR signaling pathway in order to inhibit cell proliferation, invasion, migration, and epithelial-mesenchymal transition, and to exert an anti-tumor effect (Ma et al. 2020). Bai et al. experimentally confirmed that the effects of larotrectinib (Lar) was able to inhibit the proliferation and migration of CRC cells, and the underlying mechanism was related to the activation of AMPK/mTOR signaling pathway, thus inducing intracellular autophagy (Bo et al., 2024). In addition, Liu et al. showed that atorvastatin calcium (ATO) inhibited the proliferation and migration of HCT116 cells, and the mechanism may be related to the induction of cellular autophagy by regulating the protein expression of LC3 (Liu L. et al., 2024). It has been reported that dexmedetomidine-treated CRC cells have increased LC3 sites and lysosomal structures to induce autophagy and inhibit the proliferation of CRC, which in turn affects the progression of CRC (Hou and Xu, 2024). Therefore, we can find that these target protein molecules are involved in the regulation of autophagy in CRC cells and become promising options for the treatment of CRC.

#### 2.2.1 Regulation of natural products of autophagy intervention in CRC

**FIGURE 3:**
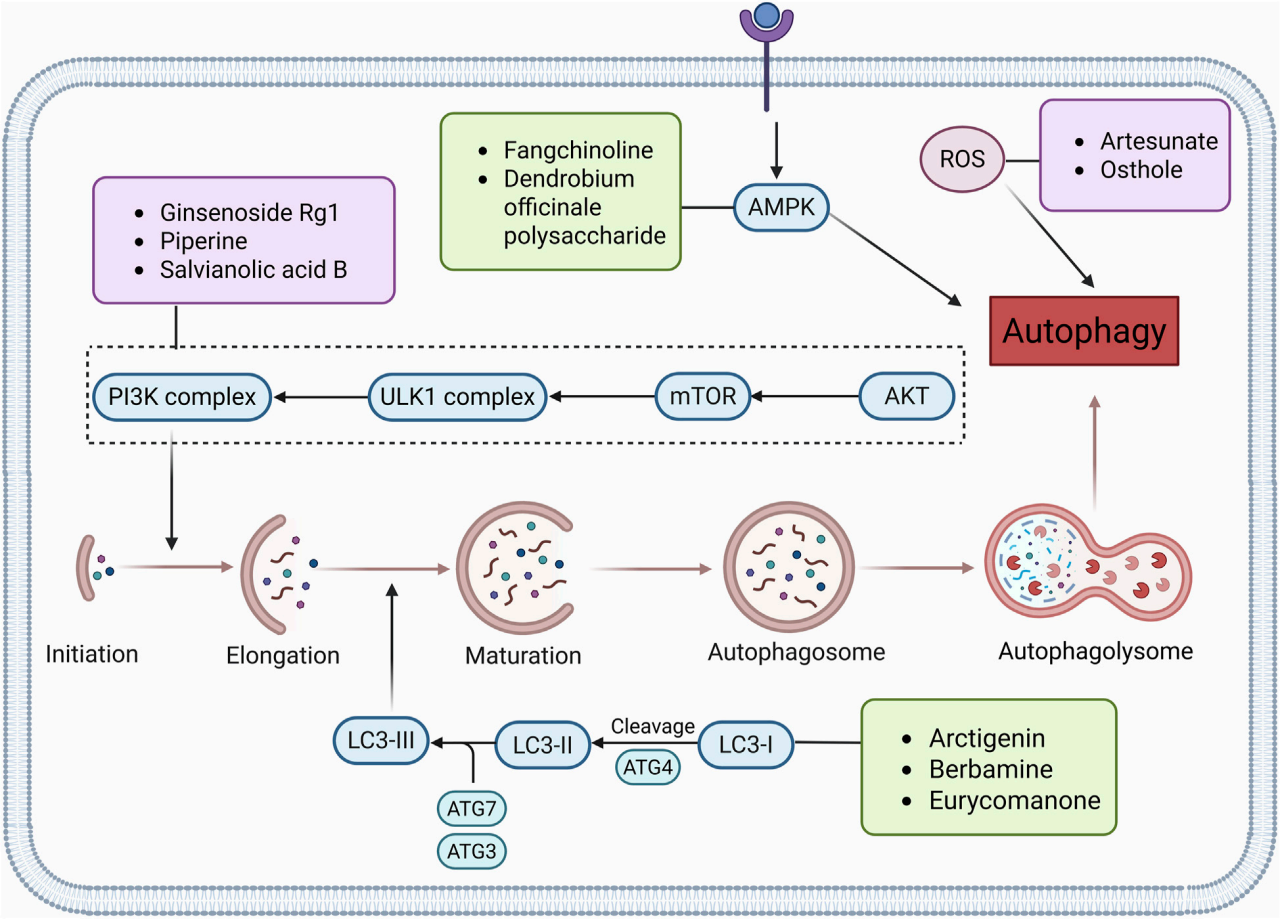
Natural products target ROS, PI3K/AKT, AMPK, and LC3 signaling pathways to regulate autophagy and play a role in intervening in CRC. Created with Biorender.com.

Autophagy plays a decisive role in all stages of human tumorigenesis, guiding tumorigenesis and promoting tumor progression and metastatic ability. Autophagy regulation is increasingly becoming a new way to treat CRC, and the following products will provide a scientific basis for this (Figure 3).

##### 2.2.1.1 Targeting the ROS signaling pathway

As mentioned above, natural products targeting ROS can regulate apoptosis to inhibit CRC tumor progression. Excitingly, targeting ROS to induce cellular autophagy also exerts such an inhibitory effect.

Artesunate, a derivative extracted from the above-ground part of *Artemisia annua L.*, family Asteraceae, has been reported to possess antioxidant and antitumor pharmacological properties (Kong et al., 2019). Huang et al. experimentally confirmed that Artesunate inhibited CRC cells by regulating the ROS-related signaling pathway and inducing cellular autophagy. In SW480 and HCT116 cells, Artesunate (1, 2, 4, and 8 μM) treatment led to mitochondrial dysfunction, greatly promoted mitochondrial ROS production, upregulated the expression of p-IRE1α, LC3B, Beclin1, and Atg3, and led to the cell cycle arrest in G0/G1 phase to inhibit cell proliferation. In in vivo experiments, the results similarly demonstrated the ability of Artesunate (30 and 60 mg/kg) to significantly inhibit tumor size and cell viability and proliferation in CT26-derived tumor model mice *in vivo* (Huang et al., 2022). In this experiment, changes in body weight and organ indices in mice indicated only low toxicity, but no long-term toxicity data were provided, limiting the drug safety assessment.

Osthole is derived from the umbelliferae plant drug *serpentine Cnidium monnieri (L.) Cuss.* was shown to have anti-tumor activity. (Sun et al., 2021). Song et al. found in Osthole intervention in CRC model mice that Osthole (50 mg/kg) induced autophagic flux damage leading to CRC cell death, reduced the number and size of tumor cells and enhanced chemotherapeutic 5-FU sensitivity. In addition, Osthole (0, 5, 10, 20, and 50 μM) significantly upregulated the levels of ROS and Beclin1, downregulated the ratio of LC3B-I/LC3B-II, disrupted mitochondrial homeostasis, decreased ATP production, and increased the level of calcium ions in HCT116 and HT29 cells, effectively inhibiting CRC cell viability and proliferation (Song et al., 2024). This study did not investigate the synergistic mechanism of Osthole with existing drugs (e.g., 5-FU) and only reported no significant synergistic effect.

Table 2 lists other natural products that inhibit CRC progression by modulating ROS-related signaling pathway-induced cellular autophagy. These results suggest that the modulation of autophagy-related targets can effectively inhibit the viability of CRC cells and tumor growth, and may become a new avenue for CRC treatment in the future.

**TABLE 2:** Natural compounds target autophagy signaling pathway to treat CRC.

Extract	Origination	Structure	Models	Biological effects	Results	References
Artesunate	*Artemisia annua L*	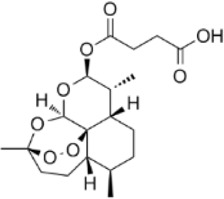	SW480/HCT116 Cells CRC model mice	p-IRE1α↑LC3B↑Beclin1↑Atg3↑ROS↑	Induce cellular autophagy G0/G1 cell cycle arrest Inhibit cell proliferation	Huang et al. (2022)
Osthol	*serpentine Cnidium monnieri (L.) Cuss*	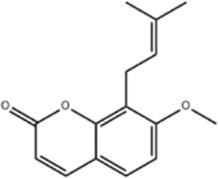	HCT116/HT29 Cells CRC model mice	ROS↑Beclin1↑ LC3B-I/LC3B-II↓ ROS↑	CRC cell death Reduce the number and size of tumor cells Enhance sensitivity to the chemotherapeutic agent 5-FU	Song et al. (2024)
Xanthatin	*Xanthium strumarium L*	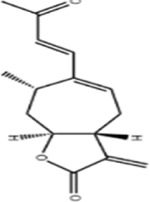	HT29 Cells	ROS↑Beclin1↑ LC3B-I/LC3B-II↓ ROS↑	Inhibit the proliferation of human CRC	Geng et al. (2020)
Ginsenoside Rg1	*ginseng Panax ginseng C.A. Mey*	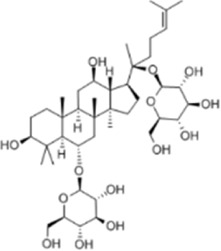	CT26 Cells CRC model mice	LC3↑ Beclin-1↑p-Akt↓p-mTOR↓p-p70S6k↓	Inhibit tumor growth and proliferation	Liu L. et al. (2024)
Piperine	*Piper nigrum*	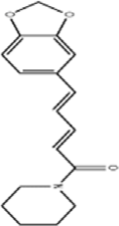	SW480/HCT116 Cells CRC model mice	LC3↑LC3-II↑ROS↑Beclin1↑p-AKT↓mTOR↓	Suppress tumor volume and weight	Xia et al. (2024)
Salvianolic acid B	*Salvia miltiorrhiza Bge*	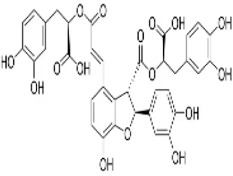	HCT116/HT29 Cells CRC model mice	LC3-II↑Atg5↑AKT↓mTOR↓	Inhibit cell viability and proliferation Reduce colony number and size	Jing et al. (2016)
Polyphyllin II	*Rhizoma Paridis*	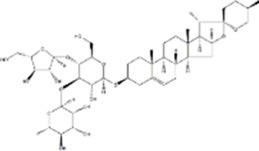	HCT116/SW620 Cells CRC model mice	LC3B-II↑PI3K↓mTOR↓	Inhibit cell viability and proliferation	Li et al. (2022)
Scoparone	*Artemisia capillaris Thunb*	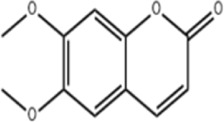	HT29/HCT116 Cells CRC model mice	LC3-II↑PI3K↓mTOR↓p62↓	Inhibit the activity and proliferative capacity of CRC cells	Huang et al. (2023)
Myricetin	*Geum aleppicum Jacq*	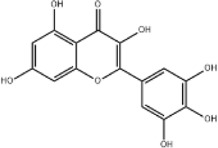	SW620/HCT116 Cells	LC3-II↑PI3K↓mTOR↓ Beclin1↑	Inhibit the activity and proliferative capacity of CRC cells	Zhu et al. (2020)
Hesperidin	*Citrus reticulata Blanco*	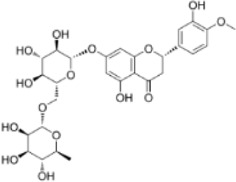	CRC model mice	LC3-II↑PI3K↓mTOR↓Beclin1↑CEA↓	Induce autophagy and inhibition of tumorigenesis	Saiprasad et al. (2014)
Polyphyllin I	*Paris yunnanensis Franch*	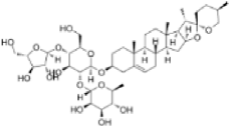	SW480 Cells	LC3-II↑PI3K↓mTOR↓ Beclin1↑	Promote autophagic cell death in CRC cells	Luo et al. (2022)
Fangchinoline	*Stephaniae tetrandine S. Moore*	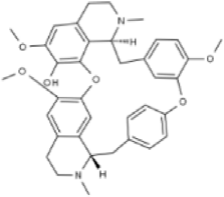	HT29/HCT116 Cells CRC model mice	LC3-II↑AMPK↑P62↓mTOR↓	Reduce volume and weight of tumors Inhibit the progression of CRC	Xiang et al. (2021)
Dendrobium officinale polysaccharide	*Dendrobium officinale Kimura & Migo*	------	CT26 Cells	LC3-II↑AMPK↑P62↓mTOR↓	Reduce the volume and weight of tumors Inhibit the progression of CRC	Zhang et al. (2021)
Arctigenin	*Fructus Arctii*	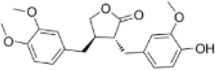	SW480/SW620 Cells	LC3-II↑AMPK↑P62	Modulation of cellular autophagy to enhance sensitivity of cisplatin-resistant CRC cells	Wang et al. (2018)
Berbamine	*Berberis amurensis Rupr*	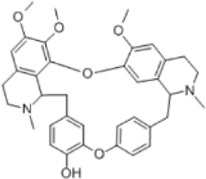	HT-29 Cells	LC3B-I↑ATG-5↑ATG-12↑ Beclin-1↑	Modulation of cellular autophagy to inhibit CRC cell activity and proliferative capacity	Mou et al. (2019)
Eurycomanone	*Eurycoma longifolia Jack*	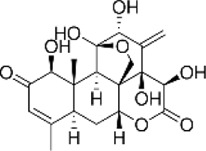	HCT116 Cells CRC model mice	ROS↓Beclin-1↓LC3-II↓GSH↑P62↑	Reduce tumor weight and size	Ye et al. (2022)
Vitexin	*Vitex negundo var. negundo*	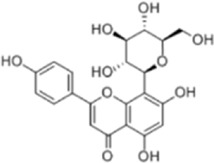	HCT116 Cells CRC model mice	ROS↓Beclin-1↓LC3-II↓	Inhibit cellular autophagy Reduce chemotherapeutic drug resistance	Bhardwaj et al. (2017)

##### 2.2.1.2 Targeting the mTOR/PI3K/AKT signaling

mTOR/PI3K/AKT is a major regulator of cellular metabolism and plays an important role in treating the course of CRC through multiple signaling pathways in order to regulate cell growth and proliferation. The following natural products can better respond to it.

Ginsenoside Rg1 is a triterpenoid saponin compound from the dried roots and rhizomes of the *Panax ginseng C.A. Mey.* from the family Wujiaceae, with excellent pharmacological properties such as anti-inflammatory, immunomodulatory, and so on (Cheng et al., 2022). Liu et al. used Ginsenoside Rg1 (100 mg/kg) to intervene in CRC xenograft model mice for 14 days. 5-FU (20 mg/kg) was used as a positive control group. Western blot results showed that Ginsenoside Rg1 upregulated the levels of autophagy marker proteins LC3 and Beclin-1, and downregulated the p-Akt, p-mTOR and p-p70S6k protein expression. Meanwhile, Ginsenoside Rg1 inhibited tumor growth and proliferation in CRC model mice *in vivo*. In addition, this experiment also revealed that Ginsenoside Rg1 (80, 160, and 320 μmol/L) was able to inhibit the viability of CT260 cells through Ginsenoside Rg1-induced CT26 cell line, and further indicated that Ginsenoside Rg1 exerted its antitumor effects through the Akt/mTOR/p70S6K signaling pathway to induce cell autophagy (Liu R. et al., 2024). This study was based on the mouse CT26 cell line and a nude mouse model and did not include human colorectal cancer cells or clinical samples for validation. In addition, the pharmacokinetics (e.g., bioavailability, metabolites) of Rg1 were not addressed, which may affect the assessment of its feasibility as a drug candidate.

Piperine, an alkaloid molecule extracted from *Piper nigrum L.* and *Piper longum L.*, has been shown to have pharmacological activities such as anti-inflammatory and anticancer (Haq et al., 2021). Xia et al. intervened in CRC xenografts by Piperine (20 mg/kg) in mice for 27 days without a positive control group. The results of the study showed that Piperine was able to inhibit the volume and weight of tumors *in vivo* in CRC model mice, increased the levels of LC3, LC3-II, ROS, and Beclin1, and decreased the levels of p-AKT and mTOR. It was further observed by HCT116 and SW480 *in vitro* experiments that Piperine (0, 25, 50, 100, and 150 μM) inhibited CRC cell viability and proliferative capacity in a dose-dependent manner (Xia et al., 2024). The low water solubility and *in vivo* pharmacokinetic properties of piperine were not systematically studied in this experiment, which may limit its practical application. In addition, no coadministration experiments with existing chemotherapeutic agents (e.g., 5-FU) have been conducted to assess the potential for synergistic effects.

Salvianolic acid B (Sal B) is an active compound in the dried roots and rhizomes of *Salvia miltiorrhiza Bge*. Modern pharmacological studies have shown that Sal B has anti-inflammatory and anti-tumor properties (Fu et al., 2024). *In vitro* experiments with HCT116 and HT29 cells showed that Sal B was dose-dependent cytotoxic at a concentration of 200 μM. Mechanistically, Sal B inhibited the AKT/mTOR signaling axis and decreased the phosphorylation of AKT, mTOR and p70S6K, while activating the phosphorylation of ULK1. Intraperitoneal injection of 80 mg/kg significantly reduced tumor growth without systemic toxicity. Co-administration with the autophagy inhibitor 3-MA (20 mg/kg) attenuated anti-tumor activity, confirming the pro-death effect of autophagy (Jing et al., 2016). Despite these findings, this study did not explore the effects of Sal B in a metastatic CRC model, nor did it assess its synergistic effects with conventional chemotherapy such as 5-FU or OXA.

Polyphyllin II (PPII), a natural steroidal saponin present in *Paris yunnanensis Franch.*, has been shown to have potent anticancer activity against a wide range of cancers (Jiao et al., 2022). Li et al. showed that PPII (1, 2, 3, and 4 μM) inhibited the proliferation of colorectal cancer cells, induced G2/M-phase cycle blockade, and activated autophagy process in a dose-dependent manner. The mechanism includes downregulation of PAK1, blocking of AKT/mTOR signaling pathway, resulting in increased levels of LC3B-II and decreased phosphorylation of PI3K/AKT/mTOR pathway. Experiments in xenografted CRC mice showed that PPII (50 mg/kg/day) significantly inhibited the growth of transplanted tumors without significant toxicity (Li et al., 2022). Although the current study reveals the mechanism by which PPII exerts antitumor effects through autophagy regulation, further optimization of *in vivo* delivery strategies and exploration of its combination therapy with targeted drugs are needed.

Scoparone (Scop) is a natural bioactive substance extracted from the plant drug *Artemisia capillaris Thunb*, which has been proved to have anti-inflammatory and anticancer properties in modern pharmacology (Liu Y. et al., 2020). Huang et al. explored the mechanism of action of Scop on CRC cells by HT29 and HCT116 cell lines. The results showed that Scop (0, 100, 200, and 300 μM) inhibited the activity and proliferation of CRC cells in a dose-dependent manner, and western blot showed that Scop upregulated the expression of LC3-II, ATG5, and Beclin1, and lowered the levels of P62, PAK1, AKT, and mTOR, which was related to the inhibition of AKT/mTOR signaling pathway to promote the cell proliferation. mTOR signaling pathway to promote cellular autophagy. In addition, Scop (50 mg/kg) was able to significantly inhibit the volume and weight of tumors in xenograft CRC model mice *in vivo* (Huang et al., 2023). However, autophagosome formation was not directly observed by electron microscopy in this study, which may affect the complete assessment of autophagic flow. In addition, the experiment of cell viability recovery after inhibition of autophagy did not exclude the interference of other cell death pathways (e.g., apoptosis), which needs to be further verified.

Table 2 lists other natural products that inhibit CRC progression by modulating cellular autophagy induced by the mTOR/PI3K/AKT-related signaling pathway. Thus, mTOR/PI3K/AKT becomes a potential target for natural products that inhibit CRC tumor growth.

##### 2.2.1.3 Targeting the AMPK signaling pathway

AMPK is a key regulator of energy metabolism and plays a critical role in the control of cell growth and regulation of cellular autophagy. The results of several studies have demonstrated the ability of natural products to modulate AMPK signaling, thereby affecting the prevention and management of CRC.

Dendrobium officinale polysaccharide (DOP) is an active compound extracted from orchid medicines, and modern pharmacological studies have demonstrated the antitumor effects of DOP. Modern pharmacological studies have demonstrated that DOP has significant anti-tumor effects (Wang et al., 2022). Zhang et al. intervened CT26 cells using DOP (200, 400, and 800 μg/mL) to elucidate the mechanism of action. The results showed that DOP inhibited the proliferation of CT26 and HCT116 cells in a dose-dependent manner in the concentration range of 0–800 μg/mL, induced ROS generation, disrupted MMP, and inhibited ATP production, which in turn activated the AMPK/mTOR pathway. DOP-induced cytotoxicity, excessive autophagy and mitochondrial dysfunction were reversed after ROS removal using NAC in the experiments, validating the critical role of ROS in this process (Zhang et al., 2021). No *in vivo* experiments were performed in this study, so the efficacy and safety of DOP *in vivo* could not be fully assessed.

##### 2.2.1.4 Targeting the LC3-related protein signaling pathway

LC3-associated protein is a marker of autophagosome formation, and its dysregulation is inextricably linked to the progression of a variety of diseases, while it has also become the centerpiece of mammalian autophagy monitoring. Some studies have found that regulation of autophagy-related protein LC3 can inhibit CRC progression. Fortunately, we found them in natural products.

Berbamine (BE), a quinoline alkaloid extracted from *Berberis vulgaris L*., possesses a variety of pharmacological activities, including anti-inflammatory and anticancer (Farooqi et al., 2022). Mou et al. treated the HT29 cell line with different doses of BE (0, 7, 14, and 28 μM) to pass CCK8, Western Blot, and wound healing assays to analyze the action mechanism of BE on CRC. The experimental results showed that BE significantly inhibited the migration of HT29 cells at a concentration of just 14 μM, and Western blot detected the upregulation of Bax and Caspase-3/9 expression, the downregulation of Bcl-2 expression, and a significant increase in the expression of LC3B-II, ATG5, ATG12, and Beclin-1 (Mou et al., 2019) This study provides a theoretical basis for BE as a potential therapeutic agent for CRC, but its limitations are the lack of *in vivo* animal experimental validation and the unexplored differences in the effects of this drug in other colon cancer cell lines.

Eurycomanone (EN) is a diterpenoid extracted from *Eurycoma longifolia Jack*, a natural medicine widely distributed in Southeast Asia, and modern pharmacological research studies have shown that EN has anti-inflammatory and anti-tumor properties (Xu et al., 2024). Ye et al. used EN (0, 8, 16, 24, and 32 μM) to induce HCT116, SW620 cell lines. The results showed that the IC_50_ of EN on HCT116, SW620 and other CRC cell lines was 20.9–35.8 μM, which significantly reduced cell viability, clone formation ability and migration ability, and decreased the phosphorylation level of vascular endothelial growth factor receptor 2 (VEGFR2). *In vivo* experiments showed that 10 mg/kg of EN inhibited autophagy through activation of the mTOR signaling pathway, reduced tumor volume by 58.8%, downregulated the expression of the proliferation marker Ki-67, upregulated the level of the autophagy substrate p62, and inhibited vascular endothelial growth factor (VEGF)-induced angiogenesis, reducing lumen formation and endothelial cell migration (Ye et al., 2022). However, this study still has some shortcomings, such as the lack of validation of the efficacy of EN in other solid tumors.

Table 2 lists other natural products. Thus, modulating autophagy by targeting LC3-related signaling pathways offers a promising strategy for CRC treatment.

### 2.3 Ferroptosis

Ferroptosis is a type of cell death induced by lipid peroxidation of highly expressed unsaturated fatty acids in the cell membrane catalyzed by divalent iron or ester oxygenase (Zeng et al., 2023). The main features can be characterized as follows: ① Iron-dependent: the occurrence of ferroptosis is dependent on the accumulation of intracellular iron, which leads to a decrease in glutathione peroxidase activity and an increase in lipid peroxidation through the Fenton reaction; ②Lipid peroxidation: lipid peroxidation of unsaturated fatty acids occurs in response to divalent iron or esteroxygenase; ③Impairment of the antioxidant system: Glutathione peroxidase 4 (GPX4), as the core enzyme of the antioxidant system, its activity is reduced in ferroptosis, leading to a decrease in cellular antioxidant capacity; ④ cell death: cell death occurs as a result of lipid peroxidation and impaired antioxidant system (Mou et al., 2019). Several data support that the occurrence of Ferroptosis is closely related with the expression of GPX4, NFE2-related factor 2 (Nrf2) and other key signaling molecules (Liu X. et al., 2022). GPX4, a monomeric glutathione peroxidase with a catalytic role in the reduction of lipid peroxides, plays an important role in the process of ferroptosis and protects cells and tissues from free radicals damage by inhibiting lipid peroxidation. In contrast, deletion or dysfunction of GPX4 can lead to the accumulation of intracellular peroxides, which induces the onset of cellular ferroptosis and causes cell death (Liang et al., 2023). Nrf2 is considered to be a major regulator of antioxidant responses, and many pathological conditions are associated with an imbalance in redox homeostasis. In addition, Nrf2 levels are directly correlated with ferroptosis sensitivity, and upregulation of Nrf2 expression inhibits ferroptosis, whereas decreasing Nrf2 levels enhances the sensitivity of cancer cells to pro-ferroptosis agents (Dodson et al., 2019).

Notably, a large number of studies have validated the critical role of ferroptosis in inhibiting CRC progression, including the inhibition of tumor proliferation and metastasis (Wang et al., 2022). Therefore, ferroptosis is considered to be an important tumor suppressor mechanism and a potential target for CRC therapy. Sui et al. demonstrated through cell experiment that ferroptosis inducers upregulated the expression of ROS in HCT116, LoVo, and HT29 cells, and at the same time, reduced the level of GPX4 in order to inhibit the activity and proliferation of CRC cells, and exerted anti-tumor effect (Sui et al., 2018). In an experiment with CRC xenograft model mice, cetuximab enhanced RSL3-induced ferroptosis by inhibiting the Nrf2/HO-1 axis to suppress the weight and volume of CRC tumor cells, which has potential positive significance for the treatment of CRC (Yang et al., 2021). The above studies suggest that ferroptosis has great potential in the treatment of CRC, thus providing a new avenue for the treatment of CRC.

#### 2.3.1 Natural products regulating ferroptosis intervention in CRC

**FIGURE 4:**
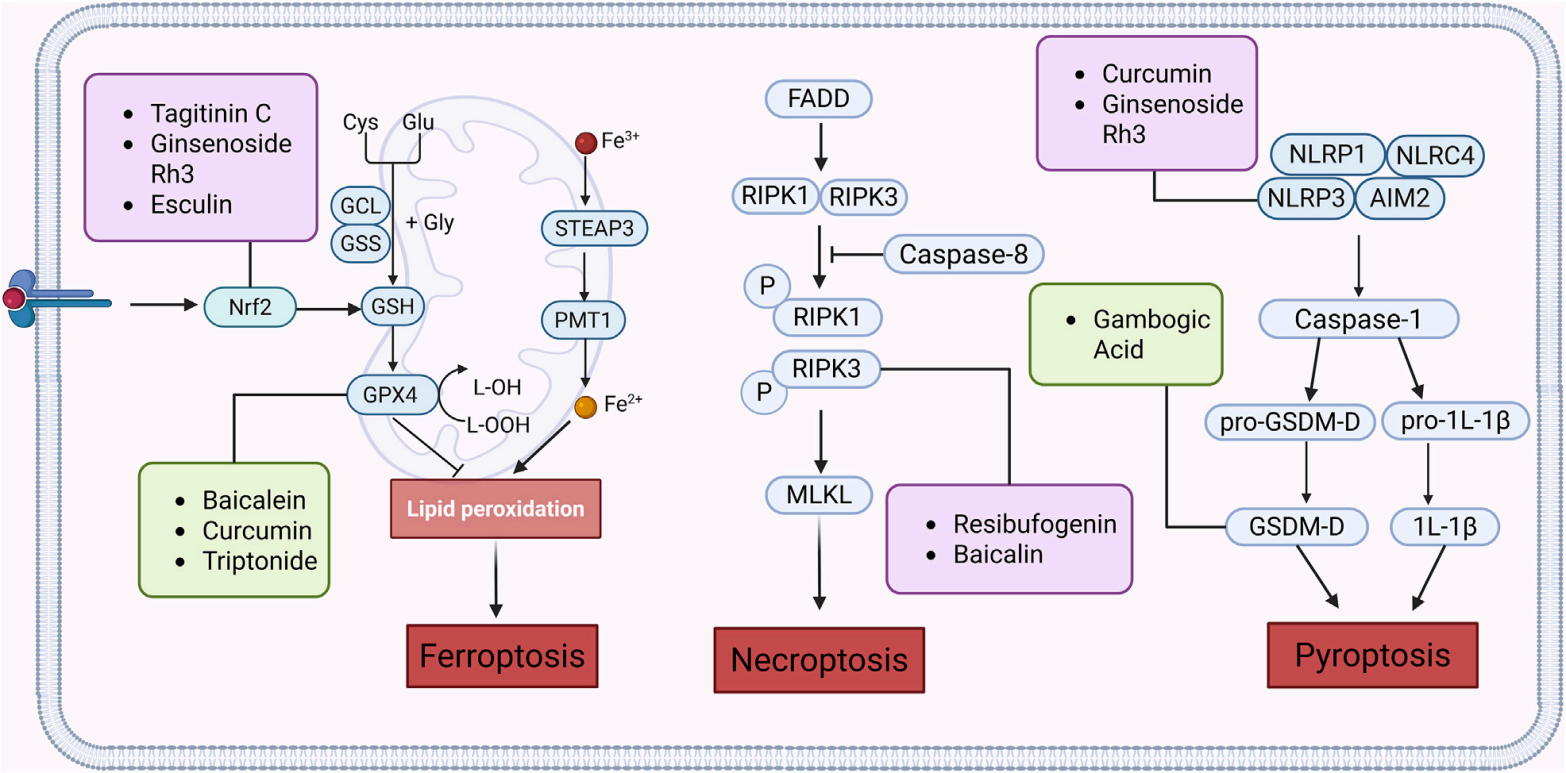
Natural products play a role in intervening in CRC by targeting signalling pathways such as Nrf2, GPX4, NLRP3, RIPK1, and RIPK3 and regulating different forms of cell death mechanisms such as Ferroptosis, Pyroptosis and Necroptosis. Created with Biorender.com.

A large amount of scientific evidence has proved that Ferroptosis plays an important role in the occurrence and development of CRC, and it may be an effective way to treat CRC. The following products have demonstrated the therapeutic effects of CRC by targeting and regulating important signaling pathways related to Ferroptosis (Figure 4).

##### 2.3.1.1 Targeting the Nrf2 signaling pathway

Nrf2 is considered to be a major cellular defense mechanism and an important regulator of cell survival, protecting cells and tissues from various toxicants and carcinogens by increasing the expression of many cytoprotective genes. Some studies have shown that targeting and regulating the Nrf2 signaling pathway by natural products has positive implications for the treatment of CRC.

Ginsenoside Rh3 (GRh3) is an active compound extracted from the dried roots and rhizomes of the plant drug *P. ginseng C.A. Mey.* Which has been shown to possess a wide range of benefits including anti-inflammatory, antioxidant and anticarcinogenic properties (Yang et al., 2022). Wu et al. found that GRh3 had a dose-dependent inhibitory effect on the proliferation of HT29, HCT116 and other CRC cells in the concentration range of 10–160 μM. In in vivo experiments, GRh3 (20 mg/kg/d) was administered by gavage, and the tumor volume of the administered mice was reduced by more than 40% compared with that of the control group after 21 days, and there was no obvious liver or kidney toxicity. Mechanistic studies confirmed that GRh3 induces thermal apoptosis and ferroptosis through the Stat3/p53/Nrf2 signaling axis (Wu et al., 2023). This trial did not explore the potential effects of long-term dosing on normal tissues and lacked studies on the combined effects with clinically used chemotherapeutic agents.

Esculin is the main active ingredient in the stem bark of *Fraxinus chinensis subsp. rhynchophylla (Hance) A.E.Murray.*, which has a variety of pharmacological effects, including anti-inflammatory, antioxidant, and antitumor (Cai and Cai, 2023). Ji et al. used Esculin (20, 40, 80 μM) to treat HCT116 cell line. CCK-8 assay and Annexin V/PI staining showed that Esculin inhibited cell viability and proliferation in a dose-dependent manner, upregulated the protein levels of Malondialdehyde (MDA), ROS, PKR, PERK, p-eIF2α, COX2 and ACSL4, and downregulated the expression of GSH, SOD and FTH1. In addition, an AOM/DSS-induced *in vivo* CRC mouse model was used to validate the potential of Esculin to inhibit tumorigenesis and to elucidate its underlying mechanisms. The experimental results further suggested that Esculin (20 and 40 mg/kg) may inhibit CRC and progression by inducing apoptosis and ferroptosis *in vivo* (Ji et al., 2024). Survival analysis was not performed in this study, and efficacy was assessed only by tumor number and colon length, lacking direct evidence of survival benefit.

Table 3 lists other natural products that inhibit CRC progression by modulating ferroptosis induced by Nrf2-related signaling pathways. The above studies suggest that modulation of Nrf2 signaling by these products is considered an effective strategy to inhibit CRC progression.

**TABLE 3:** Natural compounds target other signaling pathway to treat CRC.

Extract	Origination	Structure	Models	Biological effects	Results	References
Ginsenoside Rh3	*Panax ginseng C.A. Mey*	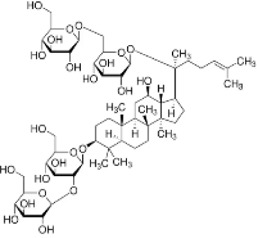	HT29/HCT116 Cells CRC model mice	MDA↑ROS↑HO-1↑SOD↓GSH↓Nrf2↑	Inhibit cell migration, viability, and proliferation	Wu et al. (2023)
Esculin	*Fraxinus rhynchophylla Hance*	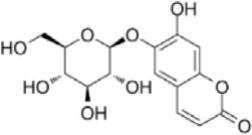	HCT116 Cells CRC model mice	MDA↑ROS↑SOD↓GSH↓FTHI↓PKR↑COX2↑	Induce apoptosis and iron death Inhibit colon carcinogenesis and progression	Ji et al. (2024)
Lysionotin	*Flemingia fluminalis C.B.Clarke ex Prain*	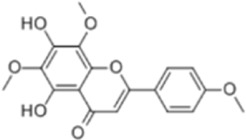	SW480/HCT116 Cells CRC model mice	MDA↑ROS↑SOD↓GSH↓Nrf2↑	Induce cellular iron death for anticancer effects	Gao et al. (2022)
Curcumin	*Curcuma longa L*	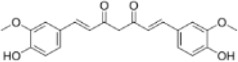	SW620/LoVo Cells CRC model mice	ROS↑MDA↑Fe2^+^↑GSH↓GPX4↓	Inhibit tumor growth Reduce tumor cell activity and proliferative capacity	Ming et al. (2024)
Baicalein	*Scutellaria baicalensis Georgi*	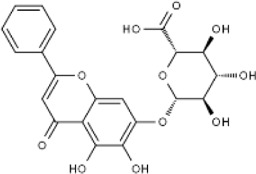	HCT116/DLD1 Cells CRC model mice	ROS↑MDA↑GSH↓GPX4↓	Inhibit tumor cell growth	Lai et al. (2024)
Triptonide	*Rehmannia glutinosa*	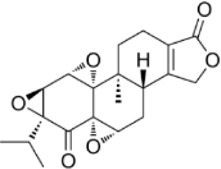	HCT116/LoVo Cells CRC model mice	ROS↑MDA↑Fe2^+^↑	Inhibit tumor cell growth	Wang et al. (2024)
Gambogic Acid	*Garcinia cambogia*	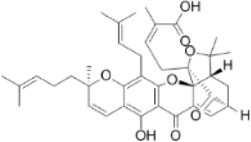	HCT116/CT26 Cells CRC model mice	Caspase-3↑ GSDME↑	Suppression of solid tumor volume and weight in CRC model mice	Xu et al. (2022)
Curcumin	*Curcuma longa.L*	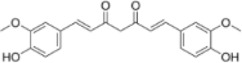	HCT116/LoVo/HT29/SW480 Cells	IL-1β↓IL-18↓NLRP3↓	Modulation of cellular focal death for anti-CRC tumor effects	Dal and Aru (2023)
Ginsenoside Rh3	*Panax ginseng C.A.Mey.*	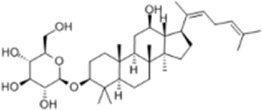	HT29/HCT116/SW620/DLD1/RKO Cells CRC model mice	IL-1β↓IL-18↓NLRP3↓	Inhibit the size and weight of tumors Reduce inflammatory infiltration	Wu et al. (2023)
Resveratrol	*Aloe vera (L.) Burm.f.*	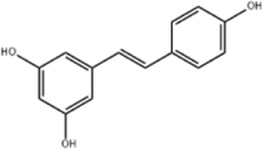	HCT116 Cells CRC model mice	IL-1β↓IL-18↓NLRP3↓ Caspase-1↓	Inhibit of cellular pyroptosis for anti-tumor effects	Ren et al. (2021)
Resibufogenin	*Ampelopsis delavayana var. glabra (Diels & Gilg) C.L.Li*	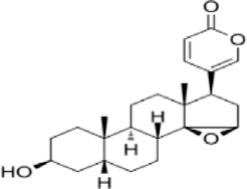	SW480/HCT-116 Cells CRC model mice	PYGL↑GLUD1↑GLUL↑ RIPK3↑ MLKL↑	Inhibit tumors *in vivo* in CRC model mice	Han et al. (2018)
Baicalin	*Scutellaria baicalensis Georgi*	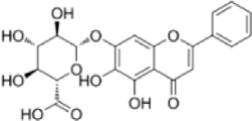	CT26.WT Cells	RIPK3↑ MLKL↑	Inhibit viability and proliferation Induce necrotic apoptosis for anti-tumor effects	Yang et al. (2019)

##### 2.3.1.2 Targeting the GPX4 signaling pathway

The GPX4 signaling pathway is an antioxidant defense system against ferroptosis in normal cells, which maintains cellular redox homeostasis and specifically converts lipid peroxides to nontoxic lipocalciferol in a GSH-dependent manner to abrogate the oxidative activity of lipid peroxides, thereby preventing cellular ferroptosis. Mediating the GPX4-related pathway has been reported to effectively intervene in CRC progression. Notably, the ability of the following natural products to inhibit CRC cell progression was achieved by modulating GPX4 targets.

As described above, Baicalein was able to mediate the NF-κB signaling pathway to induce apoptosis to inhibit the viability and proliferation of CRC cells. Excitingly, Baicalein was also able to regulate ferroptosis to inhibit the progression of CRC tumor cells. Lai et al. showed that Baicalein (7.5–30 μM) dose-dependently decreased the expression of GPX4, GSH, and upregulated the levels of ROS, MDA, leading to a decrease MMP in HCT116 and DLD1 cell lines, which ultimately led to the phenomenon of ferroptosis. Furthermore, in CRC model mice, Baicalein (10 and 20 mg/kg) inhibited tumor cell growth in a dose-dependent manner. The mechanism is related to the fact that Baicalein induces ferroptosis through inducing the JAK2/STAT3/GPX4 axis to exert anti-CRC, providing a theoretical basis for its use as a therapeutic candidate for colorectal cancer (Lai et al., 2024). However, *in vivo* experiments have only used a subcutaneous graft tumor model, which does not mimic common clinical metastases. Furthermore, the lack of combination studies with other iron-death inducers limits the assessment of their synergistic potential.

Curcumin (CUR), an active compound in the dried rhizomes of the plant drug *Curcuma longa.L.* of the ginger family, is of interest because of its anti-inflammatory, antioxidant, and immunomodulatory, antitumor properties (Kotha and Luthria, 2019). As reported by Ming, both CUR (50, 100, and 200 mg/kg) and positive control cisplatin were able to inhibit the growth of tumors in CRC model mice *in vivo*, and reduce the activity and proliferation capacity of tumor cells. In addition, PCR, Western Blot and immunohistochemical analyses showed that CUR was able to enhance the levels of ROS, MDA, Fe^2+^, and decreased the expression of GSH and GPX4 epitopes, which was the same as the results of the *in vitro* experiments of SW620 cell line induced by CUR (10, 20, 40, and 80 μM). The therapeutic mechanism may be that CUR regulates the GSH/GPX4 axis to induce ferroptosis, thus exerting an inhibitory effect on CRC (Ming et al., 2024). However, the optimal concentration experiment and the specific concentration of cisplatin were not performed.

Triptonide (TN), a diterpenoid epoxide present in the botanical drug *Rehmannia glutinosa (Gaertn.) Libosch. ex-DC*., has been shown to have a wide range of biological properties including anti-inflammatory, antioxidant, and anticancer properties (Song et al., 2023). Wang et al. showed that the inhibitory effect of TN in HCT116 and LoVo cell lines to reveal its mechanism of action. The experimental results showed that TN (25, 50, and 100 μM) inhibited the proliferation of CRC cells and acted through ferroptosis pathway. Western blot assay showed that TN dose-dependently upregulated the levels of ROS, MDA, and Fe^2+^, and decreased the epitope protein expression of GSH and GPX4. Meanwhile, *in vivo* experiments in CRC model mice revealed that TN (5 mg/kg) inhibited tumor volume and weight in heterozygous tumor-bearing mice. In addition, this effect was associated with inhibition of the SLC7A11/GPX4 axis (Wang et al., 2024). However, this experiment was only studied at the animal and cellular level, and in the future, it needs to be evaluated in clinical trials for its safety and action effects.

The above findings suggest that these results confirm the potential role of natural product-induced ferroptosis in the treatment of CRC and that more studies are needed to support this idea.

### 2.4 Pyroptosis

Pyroptosis, which occurs predominantly in phagocytes, is a programmed cellular necrosis mediated by gasdermin proteins, dependent on inflammatory caspases and GSDMs, that forms pores in the cell membrane and releases intracellular contents to cause an intense inflammatory response (Loveless et al., 2021). Cellular pyroptosis mechanisms mainly include classical and non-classical pathways. In the classical cellular pyroptosis pathway, NLR pyrin domain-containing 3 (NLRP3), NLRC4, AIM2, Pyrin and other inflammatory vesicles are activated and cleave Pro-Caspase-1 to form active Caspase-1, which can cleave GSDMD protein to form active N-terminal and C-terminal, and the N-terminal end contributes to the perforation of cell membrane and cell death. At the same time, Caspase-1 can also process pro-IL-1β to form active IL-1β, which is released extracellularly to amplify inflammatory responses.

In addition, the Caspase-4/5/11-dependent mode of cell death is referred to as nonclassical pathway cellular pyroptosis (Vasudevan et al., 2023). In the nonclassical pathway of cellular pyroptosis, LPS can directly bind to Caspase-4/5/11, and activated Caspase-4/5/11 can cleave GSDMD proteins, and the N-terminal end of GSDMD proteins can both mediate cellular pyroptosis and activate NLRP3 inflammatory vesicles to activate Caspase-1, which ultimately produces Interleukin-1β (IL-1β) and release it to the outside of the membrane, causing an inflammatory response (Li R. et al., 2023).

In tumor immunotherapy, it has been reported that inducing tumor cell pyroptosis can activate the immune system and enhance the anti-tumor effect (Rao et al., 2022). Some studies have shown that targeting and regulating cellular pyroptosis is a promising approach for the treatment of CRC (Zhou et al., 2023). The experimental results of Guan et al. showed that targeting HDAC2-mediated cellular pyroptosis could enhance the sensitivity of CRC antitumor drug therapy, reduce drug resistance, and exert antitumor effects (Guan et al., 2024). In addition, Xie et al. demonstrated *in vivo* and *ex vivo* that simvastatin could induce pyroptosis through the ROS/caspase-1/GSDMD pathway, inhibit the proliferation of CRC cells, and suppress the growth and weight of tumors, inhibiting the progression of CRC disease (Xie et al., 2023). Therefore, targeting and regulating pyroptosis-related signaling pathways to treat CRC may be an effective strategy.

#### 2.4.1 Natural products that regulate pyroptosis intervention in CRC

NLRP3 and GSDMD have received much attention for being key regulatory proteins during the pyroptosis response, and thus regulation of this target is of profound significance in the treatment of CRC. Excitingly, we found several substances in some natural products that induce GSDMD/NLRP3 to inhibit the inflammatory infiltration and progression of CRC cells (Figure 4).

Gambogic Acid (GA), an active compound secreted in the form of dried resin by the plant *Garcinia gummi-gutta (L.) N. Robson*, was demonstrated to have a significant anticancer effect (Liu B. et al., 2020). Xu et al. used GA (0–4 μmol/L) to treat HCT116 and CT26 cell lines to elucidate the potential anticancer effects. It was found that GA reduced CRC cell viability and proliferative capacity in a dose- and time-dependent manner, and the formation of pyroptotic vesicles was observed by transmission electron microscopy. In in vivo experiments, GA (2.8 mg/kg) was able to suppress the volume and weight of solid tumors in CRC model mice, and Western blot assay revealed that GA upregulated the expression of GSDME-N, suggesting that GA-mediated GSDME-dependent pyroptosis is involved in GA-triggered cell death *in vivo* (Xu et al., 2022). In this experiment, the difference in efficacy between the high-dose group and the low-dose group was significant, but the basis for dose selection was not stated. The IC_50_ of CT26 cells was 1 μmol/L *in vitro*, while the high dose was 8 mg/kg *in vivo*, so there may be a problem of the reasonableness of the dose conversion.

Strikingly, CUR not only mediates GPX4-regulated ferroptosis to inhibit CRC cell progression, but also regulates cellular pyroptosis to exert anti-cancer effects against CRC tumors. Dal et al. used CUR to evaluate the anti-cancer mechanism on four different CRC cell lines (HCT116, LoVo, HT29, and SW480). The *in vitro* results showed that CUR (1, 5, 25, 50, and 100 μM) was able to reduce cell viability and proliferation, decrease the levels of IL-1β, IL-18, as well as increase the SubG1 phase to promote apoptosis and inhibit the development of CRC cells in all tested cell lines. In addition, in terms of cellular pyroptosis, it was found that the NLRP3 inflammatory vesicle component was elevated in SW480, HCT116 cell lines, albeit to a lesser extent in the latter, and no NLRP3 inflammatory vesicle activation was observed in LoVo and HT29 cells. It is possible that this result is due to the ineffectiveness of CUR on the NLRP3 inflammatory vesicle complex in this cell line, and in the future, we should need to further investigate other inflammatory vesicle complexes to confirm the different effects of CUR on CRC (Dal and Aru, 2023).

As described above, Ginsenoside Rh3 (GRh3) has shown great therapeutic potential in anti-inflammatory, anti-tumor, etc. Wu et al. treated (HT29, HCT116, SW620, DLD1, and RKO) cell lines with GRh3 (0, 10, 20, 40, 80, and 160 μM). The experimental results showed that GRh3 was able to inhibit the viability and proliferation of all five cell types in a dose- and time-dependent manner. In addition, the experiment to further determine whether the drug could inhibit the growth of tumor cells *in vivo*, a CRC xenograft mouse model was suggested and treated with GRh3 (20 mg/kg) intervention for 21 days without a positive control group. The results showed that GRh3 could significantly inhibit the size and weight of tumors *in vivo*, and western blot assay revealed that GRh3 could inhibit the reduction of the levels of IL-1β, IL-18, NLRP3, and reduce the inflammatory infiltration, and exert anti-tumor effects. Meanwhile HE staining showed that GRh3 could significantly inhibit CRC *in vivo* and *in vitro* without damaging the organism (Wu et al., 2023). The findings would have been more adequate if the experiment could have established a positive control group and a concentration gradient.

### 2.5 Necroptosis

Necroptosis, also known as programmed necrosis, is a form of inflammatory cell death mediated and regulated by RIPKI and RIPK3 kinases (Yan et al., 2021). Under normal conditions, signaling by TNF-α, FASL, and TRAIL recruits RIPK1 kinase to a complex of inhibitors of apoptosis (cIAPs) at the plasma membrane, leading to a cascade reaction that activates NF-κB and promotes cell survival. When this process is blocked, RIPK1 forms a secondary complex with factors such as activated caspase- 8, which induces apoptosis (Tong et al., 2022). Under conditions such as cellular stress, caspase- 8 activity is inhibited, leading to the formation of necrotic vesicles, and the regulatory function of RIPK1 is switched from apoptosis to necrotic apoptosis, while RIPK3 is recruited to necrotic vesicles and subsequently phosphorylated by RIPK1. Activated RIPK3 then phosphorylates MLKL and translocates to the plasma membrane to form a pore complex. This complex disrupts membrane integrity, enhances membrane permeability, releases inflammatory mediators, and ultimately leads to cell death (Chen et al., 2022).

There is more evidence that necrotic apoptosis plays a key role in the regulation of cancer biology, including cancer development, metastasis, and immunity (Khoury et al., 2020). Moreover, necroptosis has a bidirectional regulatory effect on cancer: on the one hand, it promotes cancer metastasis and cancer progression; on the other hand, it also prevents tumor development when apoptosis is impaired (Ye et al., 2023).

The view that receptor-interacting protein kinase 3 (RIPK3) expression is reported to be crucial in CRC, as its expression may affect the response to chemotherapy and, consequently, colorectal tumorigenesis and progression (Huang et al., 2023). Bozec et al. found that CRC model mice in the presence of RIPK3 deletion were more likely to exhibit higher levels of heterotopic hyperplasia and progress to highly differentiated intramucosal adenocarcinomas, promoting tumor formation (Bozec et al., 2016). Experimental data from Chen et al. demonstrated that 5-FU mediated the PUMA/RIPK3 signaling pathway to regulate necrotic apoptosis in order to enhance tumor cell killing and anti-tumorimmune responses, thus improving CRC treatment, thereby improving the treatment of CRC (Chen et al., 2024). The above studies suggest that necroptosis regulated by RIPK3-related signaling pathway can inhibit the progression of CRC and bring new hope for the treatment of CRC.

#### 2.5.1 Natural products that regulate necro-apoptotic in CRC

Interestingly, some products have been shown to regulate RIPK1/RIPK3/MLKL-related signaling target protein molecules to inhibit necroptosis and thus inhibit the progression and metastasis of CRC cells (Figure 4).

Resibufogenin (RBG) is an active compound extracted from the venom of Bufo toad, and has been shown to have significant anti-inflammatory and anti-tumor activity in modern pharmacology (Zhang and Jian, 2024). Han et al. treated SW480 and HCT-116 cell lines with RBG (0.1, 1, 2.5, 5, and 10 μM) and found that RBG reduced the activity and proliferation of CRC cells and inhibited the migration of the cells. *In vivo* experiments further confirmed that RBG (5 and 10 mg/kg) dose-dependently inhibited the weight and volume of tumors in CRC model mice, and western blot results showed that RBG was able to upregulate the levels of PYGL, GLUD1, GLUL, RIPK3, and MLKL. In addition, this experiment also confirmed the ability of RBG to inhibit the phenomenon of liver metastasis by immunohistochemistry and fluorescence imaging (Han et al., 2018). In the present study, although a role for necrotic apoptosis was demonstrated by RIPK3 knockdown experiments, the potential contribution of other cell death pathways (e.g., ferroptosis or pyroptosis) was not completely excluded.

Yang et al. used Baicalin (100 μmol/L) to intervene in CT26.WT cells for 72 h. The experimental results showed that after Baicalin treatment, the survival rate of CT26. WT cells decreased significantly with the prolongation of the action time, and DAPI staining showed that the nuclei of the cells were densely stained and fragmented; necrotic apoptosis was observed by transmission electron microscopy, with cell swelling, mitochondrial swelling, and exudation of the contents, and necrotic apoptosis was observed. Meanwhile, baicalin significantly upregulated the expression levels of RIPK3 mRNA and protein (P < 0.01), while z-VAD-fmk attenuated this effect. Mechanistic studies revealed that RIPK3 is a key marker protein for necrotic apoptosis and plays an important role in baicalein-induced cell death (Yang et al.). However, there are still some shortcomings in this experiment, such as the experiment was only conducted *in vitro* and lacked *in vivo* experimental validation to comprehensively assess the antitumor effect and safety of baicalin in the whole animal model.

## 3 Conclusion

CRC is a common malignant tumor characterized by uncontrolled division and survival of abnormal cells in the colon or rectum. With the improvement of living standards, the incidence of CRC tends to be younger, and the morbidity and mortality rates are high. At this stage, clinical treatments for CRC include preoperative radiotherapy and systemic therapy for downstaging, endoscopic and surgical local resection, local ablative therapy for metastasis, extensive surgery for localized and metastatic disease, palliative chemotherapy, immunotherapy, and targeted therapies, etc. The above treatments can lead to an improvement in the survival of CRC patients. However, the survival of CRC patients is still threatened, and the need to further explore new therapeutic means is an urgent issue at this stage.

In recent years, natural products have received widespread attention due to their natural origin, multi-target effects, and low toxicity. Natural products from plants, marine organisms, microorganisms and other sources have demonstrated significant anti-tumor activity in a variety of cancers. A large number of studies have shown that natural products can exert anti-CRC effects by targeting the programmed death pathway to inhibit tumor cell proliferation, induce apoptosis, inhibit angiogenesis, and modulate the immune microenvironment, among other pathways. Therefore, this paper summarizes the therapeutic potential exhibited by different forms of PCD in the treatment of CRC. In addition, we found that there are fewer natural products targeting ferroptosis, pyroptosis, and necroptosis pathways, which should be further explored in the future to expand the options of natural products for the treatment of CRC.

In terms of natural products against CRC, we clearly recognize that: (1) natural products usually have complex chemical structures and are able to intervene in cancer cell proliferation, apoptosis, invasion, and metastasis through multi-targeting actions. For example, CUR can target the AMPK/mTOR pathway to induce apoptosis in order to develop the migration and proliferation ability of CRC cells. (2) The multi-target mechanism reduces the possibility of cancer cells becoming resistant to a single drug target. (3) Natural products are generally derived from plants, microorganisms or marine organisms, which are highly biocompatible and have lower toxic side effects than chemotherapeutic drugs such as 5-FU or oxaliplatin. (4) Many natural products have strong antioxidant and anti-inflammatory properties that can improve the pro-inflammatory microenvironment of CRC. Natural products can enhance the sensitivity of chemotherapeutic drugs or reverse tumor resistance. (5) Certain natural products have the potential to prevent CRC and can be used as a means of primary prevention of cancer.

Although the above illustrates the outstanding contribution of natural products in the treatment of CRC, there are still some shortcomings, such as: (1) natural products tend to have lower absorption, bioavailability and half-life. (2) Compared with conventional chemotherapeutic agents, natural products usually have weaker anticancer potency, so single use may not be sufficient for complete tumor suppression. (3) The specific mechanisms are often complex and not fully defined, which may limit their widespread clinical use. (4) Natural products may interact with other anticancer drugs or conventional medications (e.g., anticoagulants, anti-inflammatory drugs, etc.), affecting therapeutic efficacy or increasing the risk of adverse reactions. (5) Despite showing significant effects in in vitro and animal studies, there are a limited number of high-quality clinical trials for natural products and the evidence base is weak.

Of concern, their clinical application also faces many challenges, especially low bioavailability and poor solubility. The complex structure of natural products and the fact that most of them are fat-soluble and difficult to be effectively absorbed limit their clinical efficacy. Therefore, improving water solubility and bioavailability has become an important task in modern drug development. Currently, scientists are trying to improve the drug delivery system of natural products through nanotechnology, liposome encapsulation and molecular modification. For example, nanoparticle carriers can improve the solubility and stability of drugs, and chemical modifications can help to increase the solubility of drugs in the bloodstream, thereby improving bioavailability and therapeutic efficacy. These approaches open up new possibilities for the clinical application of natural products. The successful modification of camptothecin is a classic example of natural product drug development. Originally derived from the *Mappia nimmoniana (J.Graham) Byng & Stull*, camptothecin has strong antitumor activity, but its low water solubility and instability have limited its clinical application. By optimizing the molecular structure, scientists developed Irinotecan, a modified drug with significant improvements in solubility, stability and bioavailability, which is widely used in the treatment of colorectal cancer (Mollica et al., 2012; Huang et al., 2022). The transformation of camptothecin to irinotecan demonstrates the great potential of natural products in drug development, proving that structural modifications and delivery system innovations can break through the biological limitations of natural products and advance their clinical applications. This case provides valuable experience for the research and application of other natural products and opens up new directions in cancer therapy.

Future research should focus on the following areas: (1) Further explore the mechanism of action of natural compounds through multi-omics techniques (e.g., genomics, metabolomics). (2) Conduct high-quality clinical trials to verify the safety and efficacy of natural compounds. (3) Explore the use of natural compounds in combination with chemotherapeutic drugs or immunotherapy to enhance the effect of comprehensive treatment. (4) Optimize the structure of natural compounds to improve bioavailability and stability. In conclusion, natural compounds are expected to provide safe, effective and versatile therapeutic options for CRC treatment, but their development and application strategies need to be continuously optimized.

## Glossary

ALT-1: Atractylenolide I
ATG: Arctigenin
AS-IV: Astragaloside IV
ATO: atorvastatin caleium
Bax: Bcl2-Associated X
Bcl-2: B-cell lymphoma-2
BE: Berbamine
CA: Cleistanthin A
CGA: Chlorogenic acid
COP: Coptisine
CRC: Colorectal cancer
CUR: Curcumin
DOP: Dendrobium officinale polysaccharide
EESP: ethanol extract of Spica Prunellae
EN: Eurycomanone
EVO: Evodiamine
FA: Ferulic acid
Fan: Fangchinoline
GA: Gambogic Acid
GAR E: Garcinone E
GPX4: Glutathione peroxidase 4
GRh3: Ginsenoside Rh3
GSH: Glutathione
Has: Hibiscus Anthocyanins Extracts
HP: Herba patriniae
IBC: Isobavachalcone
IL-1β: Interleukin-1β
LC3: light chain 3
MDA: Malondialdehyde
MLKL: Mixed lineage kinase domain-like protein
mTOR: mammalian target of rapamycin
NF-κB: Nuclear factor kappa-B
NLRP3: NLR pyrin domain-containing 3
Nrf2: NFE2-related factor 2
Ori: Oridonin
PCD: programmed cell death
PI3K: Phosphatidylinositol 3-kinase
PPII: Polyphyllin II
PT: Podophyllotoxin
RA: Raddeanin A
RBG: Resibufogenin
RIPK: receptor-interacting protein kinase
RSV: Resveratrol
SHI: Shikonin
SOD: Superoxidedismutase
TanIIA: Tanshinone IIA
TC: Tagitinin C
TN: triptonide)
TNF-α: tumor necrosis factor-α
TRAIL: TNF-related apoptosis-inducing ligand
TSN: Toosendanin
UA: Urolithin A
